# Strontium–Alix interaction enhances exosomal miRNA selectively loading in synovial MSCs for temporomandibular joint osteoarthritis treatment

**DOI:** 10.1038/s41368-024-00329-5

**Published:** 2025-02-01

**Authors:** Wenxiu Yuan, Jiaqi Liu, Zhenzhen Zhang, Chengxinyue Ye, Xueman Zhou, Yating Yi, Yange Wu, Yijun Li, Qinlanhui Zhang, Xin Xiong, Hengyi Xiao, Jin Liu, Jun Wang

**Affiliations:** 1 State Key Laboratory of Oral Diseases & National Clinical Research Center for Oral Diseases & Department of Orthodontics, West China Hospital of Stomatology, Sichuan University, Chengdu, China; 2 Laboratory of Aging Research and Department of Geriatrics, State Key Laboratory of Biotherapy, National Clinical Research Center for Geriatrics, West China Hospital, Sichuan University, Chengdu, China; 3 Fujian Key Laboratory of Oral Diseases & Fujian Provincial Engineering Research Center of Oral Biomaterial & Stomatological Key Lab of Fujian College and University, School and Hospital of Stomatology, Fujian Medical University, Fuzhou, China

**Keywords:** Mesenchymal stem cells, Oral diseases, Cell signalling

## Abstract

The ambiguity of etiology makes temporomandibular joint osteoarthritis (TMJOA) “difficult-to-treat”. Emerging evidence underscores the therapeutic promise of exosomes in osteoarthritis management. Nonetheless, challenges such as low yields and insignificant efficacy of current exosome therapies necessitate significant advances. Addressing lower strontium (Sr) levels in arthritic synovial microenvironment, we studied the effect of Sr element on exosomes and miRNA selectively loading in synovial mesenchymal stem cells (SMSCs). Here, we developed an optimized system that boosts the yield of SMSC-derived exosomes (SMSC-EXOs) and improves their miRNA profiles with an elevated proportion of beneficial miRNAs, while reducing harmful ones by pretreating SMSCs with Sr. Compared to untreated SMSC-EXOs, Sr-pretreated SMSC-derived exosomes (Sr-SMSC-EXOs) demonstrated superior therapeutic efficacy by mitigating chondrocyte ferroptosis and reducing osteoclast-mediated joint pain in TMJOA. Our results illustrate Alix’s crucial role in Sr-triggered miRNA loading, identifying miR-143-3p as a key anti-TMJOA exosomal component. Interestingly, this system is specifically oriented towards synovium-derived stem cells. The insight into trace element-driven, site-specific miRNA selectively loading in SMSC-EXOs proposes a promising therapeutic enhancement strategy for TMJOA.

## Introduction

As a prevalent degenerative arthropathy, temporomandibular joint osteoarthritis (TMJOA) restricts functional movements and causes debilitating pain, thereby severely deteriorates patients’ life quality. However, there is hardly any curative strategy to impede TMJOA because of ambiguous etiology.^1–7^ The therapeutic potential of mesenchymal stem cell-derived exosomes (MSC-EXOs) is gaining attention, particularly because of their capacity to modulate cartilage extracellular matrix (ECM) metabolism, restore subchondral bone homeostasis and a healthy immune microenvironment, and more.^1–5,8–15^ Exosomal miRNAs have been identified as one of the critical elements involved in fighting against osteoarthritis (OA). However, the efficiency of these exosomal miRNAs in therapeutic applications could be compromised by miRNA sorting mechanisms within the MSCs. Over 100 miRNAs have been identified as functionally activated in arthritis, with some being ‘harmful’ miRNAs (e.g., miR-23a-3p, miR-146a-5p, and miR-652-3p), which exhibit regulatory actions that promote the progression of osteoarthritis and have been considered candidate biomarkers related to symptoms.^16–18^ Blocking these harmful miRNAs typically alleviates OA.^17^ Others are ‘beneficial’ miRNAs (e.g., miR-140, miR-671, and miR-214-3p), which are negatively correlated with the pathological state of OA and typically downregulated in arthritic tissues.^8,10,11^ Activation of these beneficial miRNAs often attenuates OA.^8,10^ Synovial mesenchymal stem cells (SMSCs) have been identified as a key cell population intimately associated with the progression of osteoarthritis, contributing to the exacerbation of the inflammatory microenvironment and advancement of the disease.^19,20^ Increasing evidence indicates that alterations in the synovial exosomal miRNAs play a pivotal role in the formation of pathologic microenvironment and progression of osteoarthritis.^3,21,22^ Therefore, optimizing the miRNA compositions within synovial mesenchymal stem cell-derived exosomes (SMSC-EXOs) could open new avenues to enhance their therapeutic efficacy.

The roles of trace elements (strontium, copper, selenium, manganese, zinc, etc.) in regulating miRNA composition in bone marrow-derived mesenchymal stem cells (BMSCs), human umbilical vein endothelial cells, and macrophages have been addressed recently.^13,23–28^ Among them, strontium (Sr), a bone-seeking divalent metal ion, especially merits attention due to its decreased concentration in the arthritic synovial microenvironment and the known effects of Sr ion drugs on arthritis remission.^29–32^ Sr iron drugs have been shown to decrease fracture incidences, attenuate articular cartilage erosion and soothe the pain of the joint region in osteoarthritis.^13,33–36^ However, adverse events, such as gastrointestinal disorders and increased cardiovascular risk have strongly limited the use of Sr iron drugs in long-term treatment.^30,32,33^ Recently, a scaffold containing Sr was shown to promote BMSCs to release more effective therapeutic exosomes to treat critical bone defects.^13^ Previous studies often focused on the roles of Sr in promoting osteogenesis and angiogenesis, inhibiting osteoclastogenesis, balancing ECM anabolism and catabolism, and modulating immune patterns.^13,33,34,36^ However, the effect of Sr on driving miRNA sorting in SMSCs for OA or TMJOA remains unclearly.

Alix (Apoptosis-linked gene 2 interacting protein X), ubiquitously expressed and concentrated in phagosomes and EXOs, is involved in virus entry, neurodegeneration, tumor-mediated immunosuppression and more, but rarely studied in the context of arthritis.^37–39^ In our preliminary experiment, we found that the expression of Alix was downregulated in TMJOA synovial tissues (data not shown). Alix has been shown to enhance therapeutic efficacy of iPSC-derived exosomes and control exosomal protein sorting.^40^ Previous studies also have shown that Alix participates in the assembly and release of many RNA viruses.^41–43^ These data indicate that the low level of Alix in arthritic SMSCs may be related to the progression of osteoarthritis and involved in the Sr-triggered exosomal miRNA selectively loading in SMSCs.

In this study, we explore the utilization of the trace element strontium (Sr) to refine miRNA profiles within synovial mesenchymal stem cell-derived exosomes. We propose that Sr interaction with the Alix protein plays a pivotal role in selectively enriching therapeutic miRNA profiles. Our investigation reveals Alix’s role in Sr-triggered miRNA loading, with miR-143-3p as a key exosomal component for anti-TMJOA effects, thereby providing an insight into the site-specific miRNA selectively loading and offering a promising strategy for TMJOA therapy.

## Results

### Strontium augmentation elevates the yield of SMSC-EXOs and improves miRNA profiles for effective osteoarthritis intervention

**Fig. 1:**
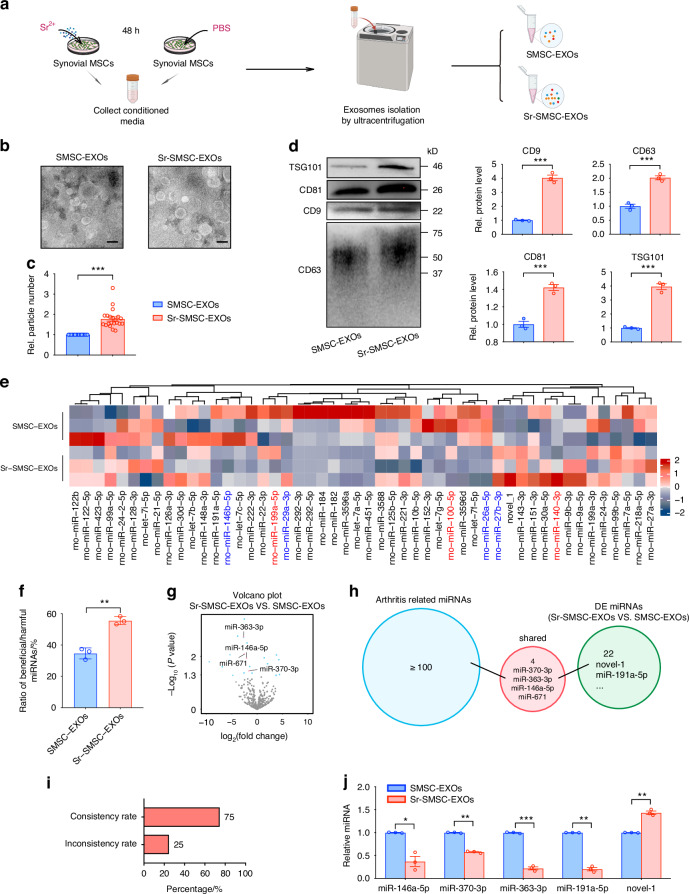
Strontium augmentation elevates the yield of SMSC-EXOs and improves miRNA profiles for effective osteoarthritis intervention. **a** Schematic diagram of untreated SMSC-EXOs and Sr-SMSC-EXOs isolation process. **b** Electron micrographs of exosomes. Scale bars, 200 nm. **c** number of particles were assessed by nanoparticle tracking analysis. **d** Western blot of marker proteins CD9, CD63, CD81, and TSG101 in exosomes. **e** Heatmap analysis of the top 50 significantly expressed miRNAs in the SMSC-EXOs and Sr-SMSC-EXOs. **f** Ratio of beneficial/harmful miRNAs (%). **g** Volcano plot of the miRNAs in the SMSC-EXOs and Sr-SMSC-EXOs. **h** Functional analysis of differentially expressed miRNAs between the SMSC-EXOs and Sr-SMSC-EXOs based on existing studies. **i** The relationship between miRNAs with known function in arthritis and DE miRNAs. **j** Quantitative RT-PCR analysis of the miRNA in the SMSC-EXOs and Sr-SMSC-EXOs. Data are represented as mean ± SEM. *P < 0.05, **P < 0.01, ***P < 0.001

To investigate the changes of overall exosomal miRNA content resulting from Sr pretreatment, the physicochemical characteristics, and the miRNA profiles of untreated SMSC-EXOs and Sr-pretreated SMSC-derived exosomes (Sr-SMSC-EXOs) were analyzed. First, we isolated exosomes from Sr-pretreated and control SMSCs (Fig. 1a, Fig. S1a-d). The features of EXOs were analyzed by transmission electron microscope (TEM), nanoparticle tracking analyzer (NTA) and western blot (WB). Both types of EXOs showed a typical bilayer teacup shape, and the diameter of the EXOs ranged from 50 nm to 150 nm (Fig. 1b, Fig. S2). As shown in Fig. 1c, the overall number of Sr-SMSC-EXOs was significantly higher than that of SMSC-EXOs. The expressions of exosome surface markers (CD9, CD63, CD81, and TSG101) in Sr-SMSC-EXOs also increased significantly (Fig. 1d). In addition, CCK-8 and EDU assays showed that Sr had no effects on the proliferation or survival of SMSCs at working concentrations (Fig. S1e, f). Thus, Sr pretreatment could boost the yield of SMSC-EXOs.

Second, the total RNA purified from SMSC-EXOs and Sr-SMSC-EXOs was used for small RNA sequencing. Heatmap analysis showed the top 50 significantly expressed miRNAs in SMSC-EXOs and Sr-SMSC-EXOs (Fig. 1e). As predicted, the miRNA pool included both beneficial and harmful miRNA components (Fig. 1e, f). Among the top 100 significantly expressed miRNAs, the ratio of beneficial/harmful miRNA in SMSC-EXO was about 1:(2.91 ± 0.33) (34.64% ± 3.53%). Interestingly, Sr pretreatment of SMSCs resulted in elevation of the ratio, which increased to 1:(1.80 ± 0.08) (55.53% ± 2.54%) (Fig. 1f). Twenty-two differentially expressed (DE) miRNAs were identified by DE Seq analysis (Fig. 1g). Among them were several ‘harmful’ (miR-370-3p, miR-363-3p and miR-146a-5p) and ‘beneficial’ (miR-671) miRNAs with previously reported roles in osteoarthritis.^10,17,44,45^ Sr-SMSC-EXOs contained less harmful miRNAs (miR-370-3p, miR-363-3p and miR-146a-5p) than SMSC-EXOs (Fig. 1h and Tab. S1). The consistency rate reached up to 75% (Fig. 1i). To verify the sequencing analysis, the expression levels of some miRNAs with known (miR-146a-5p, miR-363-3p and miR-370-3p) and unknown (miR-191a-5p and novel-1) functions in arthritis were analyzed by Quantitative Real-time PCR (qRT-PCR). Consistent with the sequencing data, the expression of miR-146a-5p, miR-363-3p, miR-370-3p and miR-191a-5p decreased while novel-1 increased (Fig. 1j). The above data suggest that treatment with Sr not only significantly elevated the yield of SMSC-EXOs, but also optimized the miRNA profile within these exosomes for effective OA intervention, selectively increasing the level of therapeutic miRNAs, while reducing the presence of potentially deleterious miRNAs.

### Superior therapeutic performance of Sr-enhanced SMSC-EXOs in ameliorating TMJOA symptoms in rats

**Fig. 2:**
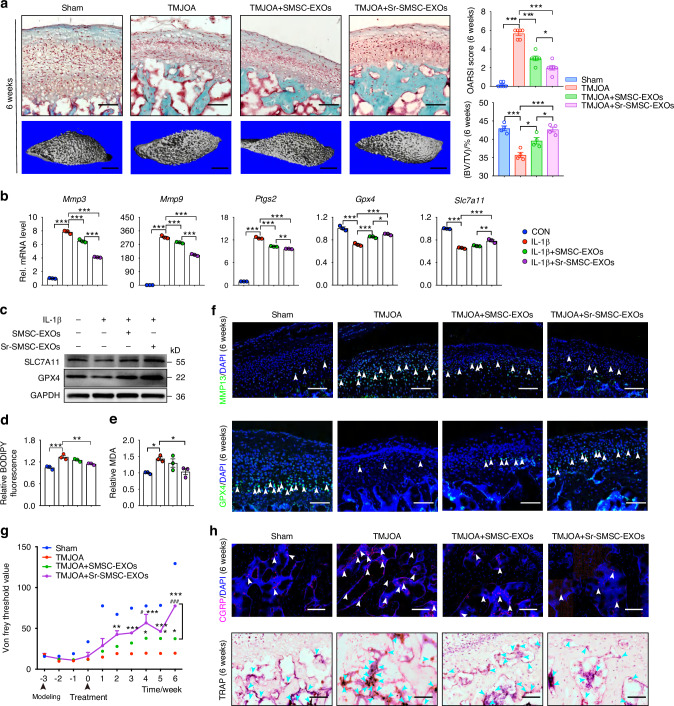
Superior therapeutic performance of Sr-enhanced SMSC-EXOs in ameliorating TMJOA symptoms in rats. **a** Safranin-O/Fast green staining, OARSI scoring system showing the degradation in condylar cartilage, and 3D reconstruction of the condyles and the ratio of bone volume to tissue volume (BV/TV) in subchondral bone. Scale bar for Safranin-O/Fast green staining, 100 μm; Scale bar for 3D reconstruction, 1 mm. **b** Quantitative RT-PCR analysis of condylar chondrocytes 48 h after indicated treatment. **c** Western blot analyses of the GPX4 and SLC7A11 in condylar chondrocytes 48 h after indicated treatment. **d** Lipid peroxidation was determined using the BODIPY 716 581/591 C11 reagent in condylar chondrocytes 48 h after indicated treatment. **e** Relative MDA measurement in condylar chondrocytes 48 h after indicated treatment. **f** Immunofluorescence staining for MMP13 and GPX4 in condylar cartilage at 6 weeks. Arrowheads indicate positive cells. Scale bar, 100 μm. **g** Measurement of the pain threshold value in TMJ region by Von Frey monofilaments testing. Compared to TMJOA group, *P < 0.05, **P < 0.01, ***P < 0.001; compared to TMJOA+SMSCs-EXOs group, ^#^*P* < 0.05, ^###^*P* < 0.001. **h** Immunofluorescence staining for CGRP and TRAP staining in subchondral bone at 6 weeks. Scale bar, 100 μm. Arrowheads indicate positive cells. Data are represented as mean ± SEM. *P < 0.05, **P < 0.01, ***P < 0.001

To investigate whether Sr-optimized SMSC-EXOs could achieved better therapeutic efficacy than conventional SMSC-EXOs, we examined the dynamic changes of cartilage degeneration and pain threshold in a rat model of temporomandibular joint osteoarthritis (TMJOA) after treatment. Intra-articular injections (IAI) of PBS, Sr-SMSC-EXOs or SMSC-EXOs were performed once a week for 3 or 6 weeks beginning 3 weeks after TMJOA induction (Fig. S3a). There was a smoother articular bone surface and higher density of subchondral bone in the Sr-SMSC-EXOs group than in the SMSC-EXOs group. Safranin-O/Fast green staining and micro-CT analysis showed that cartilage degradation was attenuated by both types of EXOs. The Osteoarthritis Research Society International (OARSI) score was significantly lower in the Sr-SMSC-EXOs group than in the SMSC-EXOs group at 3 and 6 weeks post-first injection (Fig. 2a, Fig. S3b). The bone volume/tissue volume (BV/TV) and trabecular thickness (Tb. Th) also increased in rats treated with Sr-SMSC-EXOs (Fig. 2a, Fig. S3b). Thus, compared to SMSC-EXOs, Sr-SMSC-EXOs had superior therapeutic performance on alleviating cartilage erosion.

To further explore the mechanism by which Sr-SMSC-EXOs protect cartilage from erosion better than SMSC-EXOs, we induced chondrocytes with interleukin-1β (IL-1β) to mimic an OA microenvironment in vitro. The mRNA levels of genes associated with catabolic activity (*Mmp3*, *Mmp9*, and *Ptgs2*) (Fig. 2b) in IL-1β-treated chondrocytes significantly decreased after exosome administration while anabolism-associated genes (*Col2a1* and *Acan*) were hardly affected (Fig. S3c). Compared to the SMSC-EXOs group, the levels of *Mmmp3, Mmp9, and Ptgs2* mRNA were lower in the Sr-SMSC-EXOs group. Our previous study demonstrated that ferroptosis contributes to cartilage degeneration.^46^ WB, qRT-PCR, C11-bodipy, malondialdehyde (MDA), glutathione (GSH), and Nile Red staining analysis showed that Sr-SMSC-EXOs led to higher ferroptosis inhibition with increased levels of GPX4, SLC7A11 and GSH alongside a decreased level of lipid peroxidation compared to SMSC-EXOs (Fig. 2b-e, Fig. S3d-f). In line with in vitro data, immunofluorescence staining showed that compared to SMSC-EXOs, Sr-SMSC-EXOs injection also led to elevated GPX4, decreased MMP13-positive chondrocytes in TMJOA rats (Fig. 2f, Fig. S3g). These data imply that ferroptosis inhibition might be involved in the mechanism by which Sr-SMSC-EXOs achieve enhanced therapeutic efficacy for cartilage repair.

To further compare the effects of Sr-SMSC-EXOs and SMSC-EXOs on pain relief, we first analyzed the pain threshold of TMJOA rats using a set of Von Frey monofilaments. The pain threshold gradually increased after EXOs injection in both groups. Interestingly, the pain-relieving effect of Sr-SMSC-EXOs on TMJOA rats appeared significant at 2 weeks post-first injection while that of SMSC-EXOs was delayed to 4 weeks post-first injection. The pain threshold of rats in the Sr-SMSC-EXOs group was about twice that of the SMSC-EXOs group at 6 weeks post-first injection (Fig. 2g). Cao lab’s study has revealed that the subchondral osteoclasts triggered the progression of pain via sensory innervation in arthritis.^47^ To further confirm the effect of Sr-SMSC-EXOs on pain alleviation, immunofluorescence, and tartrate resistant acid phosphatase (TRAP) staining were conducted to assess the expression of CGRP and osteoclast activity in subchondral bone. We observed that Sr-SMSC-EXOs injection reduced both markers more than SMSC-EXOs at 6 weeks post-first injection (Fig. 2h, Fig. S3h). The above data indicate that Sr-SMSC-EXOs exhibit superior pain relief compared to SMSC-EXOs.

### Necessity of Alix upregulation for Sr-triggered miRNA loading and exosome secretion

**Fig. 3:**
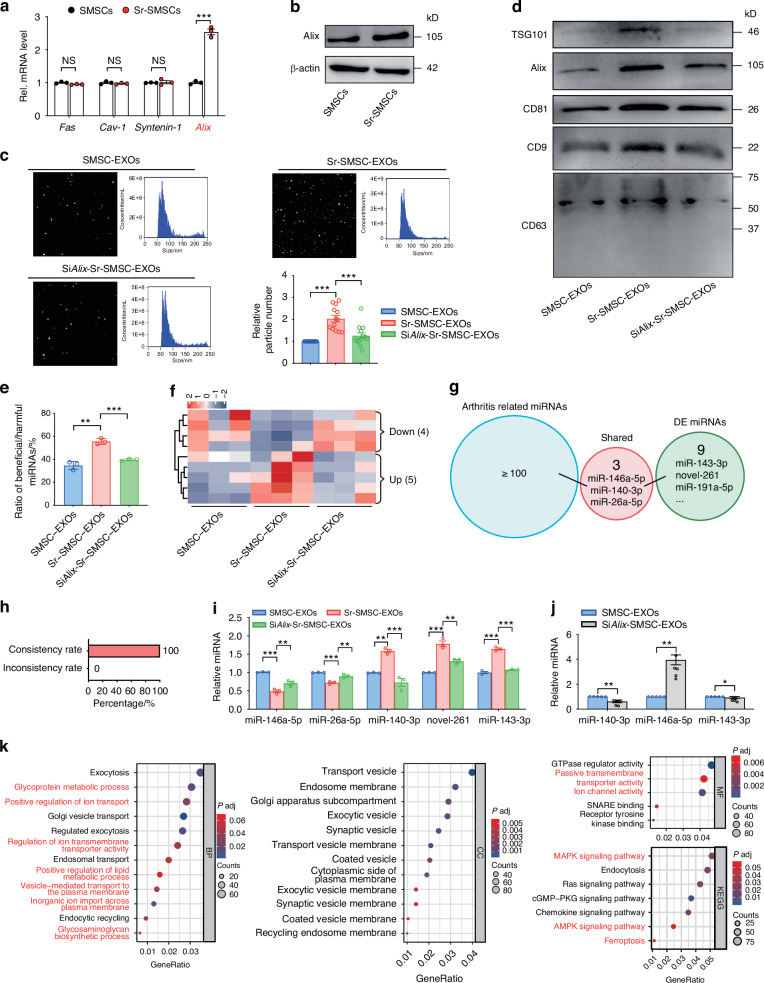
Necessity of Alix upregulation for Sr-triggered miRNA loading and exosome secretion. **a** Quantitative RT-PCR and **b** western blot analyses of exosome formation related genes in SMSCs 48 h after indicated treatment. **c** Size distribution and number of particles were assessed by nanoparticle tracking analysis. **d** Western blot of marker proteins CD9, CD63, CD81, Alix, and TSG101 in exosomes. **e** Ratio of beneficial/harmful miRNAs (%). **f** Heatmap analysis of screened miRNAs in the SMSC-EXOs and Sr-SMSC-EXOs. **g** Functional analysis of screened in miRNAs between the SMSC-EXOs and Sr-SMSC-EXOs based on existing studies. **h** The relationship between functionally known miRNAs and screened miRNAs. **i** Quantitative RT-PCR analyses of the miRNA in the SMSC-EXOs, Sr-SMSC-EXOs, and Si*Alix*-Sr-SMSC-EXOs. **j** Quantitative RT-PCR analyses of the miRNA in the SMSC-EXOs and Si*Alix*-SMSC-EXOs. **k** GO and KEGG pathway analyses of the target genes of screened miRNAs

In our preliminary experiment, immunofluorescence staining showed that the expression of Alix was downregulated in OA synovial tissues (data not shown). To further explore the precise molecular mechanism by which Sr regulates exosome biogenesis, we first examined the changes of some exosomal miRNA and protein loading-related factors (including *Alix, Fas, Cav-1, Syntenin-1, Vps28, Chmp4, Snf8, Snx*, and *Hrs*)^48–52^ in SMSCs after Sr exposure by qRT-PCR and WB (Fig. 3a, b, Fig. S4a). We found that the expression of Alix increased significantly after Sr exposure (Fig. 3a, b). Knockdown of Alix in Sr-pretreated SMSCs by siRNA transfection maintained the morphology and diameter of the exosomes (Fig. 3c, Fig. S4b-d). However, the increase in the number of nanoparticles and the expressions of exosome surface markers, such as CD9, CD63, CD81, and TSG101, were impeded (Fig. 3c, d, Fig. S4e). These findings suggest that Sr promotes SMSCs to secrete more EXOs at least partially via the upregulation of Alix.

To further investigate the role of Alix in Sr-triggered miRNA loading, we performed miRNA profile analysis of SMSC-EXOs, Sr-SMSC-EXOs and Alix siRNA knockdown (Si*Alix*)-Sr-SMSC-EXOs. Nine related miRNAs were identified. Knockdown of Alix in SMSCs abrogated the Sr-induced increase of the beneficial/harmful miRNA ratio among the top 100 significantly expressed miRNAs (Fig. 3e). Heatmap analysis showed that five Sr-elevated miRNAs were downregulated, and four Sr-decreased miRNAs were upregulated by Alix reduction (Fig. 3f). Compared with Sr-SMSC-EXOs, Si*Alix*-Sr-SMSC-EXOs contained more harmful (miR-146a-5p and miR-26a-5p) and less beneficial miRNAs (miR-140-3p). The consistency rate reached 100% (Fig. 3g, h, Tab. S2). Furthermore, the sequencing data were validated using qRT-PCR, which confirmed the differential expression of some functionally known (miR-146a-5p, miR-26a-5p and miR-140-3p) and unknown (miR-143-3p and novel-261) miRNAs (Fig. 3i). In addition, knockdown of Alix in SMSCs also led to a decrease in the abundance of miR-140-3p and miR-143-3p, coupled with an increase in miR-146a-5p (Fig. 3j). These data indicate that Sr exposure might drive SMSCs to load more beneficial and less harmful miRNA into EXOs, which is at least partially explained by the Alix dependent miRNA loading.

Finally, the biological processes (BP), cellular components (CC), molecular functions (MF), and signaling pathways regulated by Sr/Alix sorted miRNAs were determined by Gene Ontology (GO) and Kyoto Encyclopedia of Genes and Genomes (KEGG) analyses. GO analysis showed that the identified nine miRNAs are associated with ‘positive regulation of lipid metabolic process’ and ‘glycosaminoglycan biosynthetic process’. In the KEGG analysis, the miRNAs were also found to be involved in the ‘MAPK signaling pathway,’ ‘AMPK signaling pathway,’ and ‘Ferroptosis’ (Fig. 3k). These results suggest that Sr/Alix controlled miRNAs might be involved in chondrocytes ferroptosis, the inflammatory microenvironment and subchondral bone remodeling.

### Alix suppression negates Sr’s therapeutic efficacy in SMSC-EXOs for TMJOA management

**Fig. 4:**
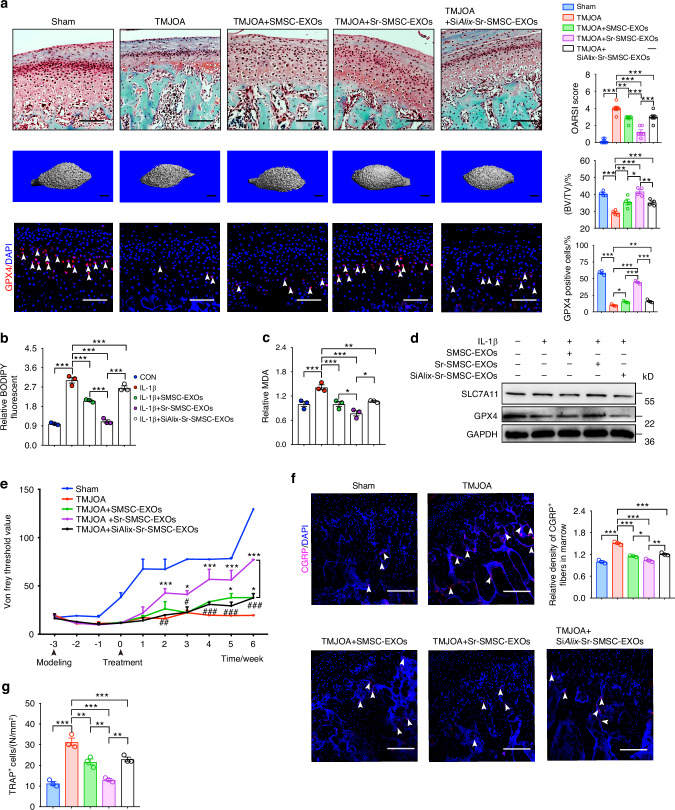
Alix suppression negates Sr’s therapeutic efficacy in SMSC-EXOs for TMJOA management. **a** Safranin-O/Fast green staining, OARSI scoring system, and immunofluorescence staining for GPX4 in condylar cartilage, and 3D reconstruction of the condyles and BV/TV of subchondral bone post indicated treatment. Arrowheads indicate positive cells. Scale bar for Safranin-O/Fast green staining and immunofluorescence staining, 100 μm; Scale bar for 3D reconstruction, 1 mm. **b** Lipid peroxidation was determined using the BODIPY 716 581/591 C11 reagent in condylar chondrocytes 48 h after indicated treatment. **c** Relative MDA measurement in condylar chondrocytes 48 h after indicated treatment. **d** Western blot analyses of the GPX4 and SLC7A11 in condylar chondrocytes 48 h after indicated treatment. **e** Measurement of the pain threshold value in TMJ region by Von Frey monofilaments testing. Compared to TMJOA group, *P < 0.05, ***P < 0.001; compared to TMJOA+Sr-SMSC-EXOs group, ^#^*P* < 0.05, ^##^*P* < 0.01, ^###^*P* < 0.001. **f** Immunofluorescence staining for CGRP in subchondral bone. Scale bar, 100 μm. Arrowheads indicate positive cells. **g** TRAP staining analyses in subchondral bone. Data are represented as mean ± SEM. *P < 0.05, **P < 0.01, ***P < 0.001

To further determine whether a suppression of Alix in SMSCs would negate the enhanced therapeutic efficacy of Sr-SMSC-EXOs in vivo, we evaluated the cartilage degeneration in TMJOA rats treated with PBS, SMSC-EXOs, Sr-SMSC-EXOs or Si*Alix*-Sr-SMSC-EXOs. The results showed that the decreased OARSI score, and increased BV/TV seen in rats treated with by Sr-SMSC-EXOs were reversed by *Alix* siRNA transfection in SMSCs. The subchondral bone in the Si*Alix*-Sr-SMSC-EXOs group displayed uneven morphology and had lower density than that of the Sr-SMSC-EXOs group. In addition, immunofluorescence staining also showed that the number of GPX4 and MMP13 positive chondrocytes in the Si*Alix*-Sr-SMSC-EXOs group sharply decreased while the number of MMP13^+^ chondrocytes increased (Fig. 4a, Fig. S5a). In line with the in vivo data, in vitro assays also showed that depletion of Alix in SMSCs led to lower inhibition of ferroptosis in Sr-SMSC-EXOs, as evidenced by decreased levels GPX4, SLC7A11 and GSH alongside increased levels of *Mmp3, Mmp13, Adamts* and lipid peroxidation (Fig. 4b-d, Fig. S5b-d). These findings indicate that the Alix elevation in SMSCs is crucial to produce highly effective Sr-SMSCs-EXOs, which protects the cartilage against erosion by inhibiting ferroptosis.

Next, we explored whether Alix downregulation in SMSCs would abrogate the enhanced pain-relieving effect of Sr-SMSC-EXOs. As shown in Fig. 4e, the differences in the pain threshold between the Si*Alix*-Sr-SMSC-EXOs and Sr-SMSC-EXOs groups appeared at the beginning of EXOs injection and became significant at 2 weeks post-first injection. Consistently, the density of CGRP^+^ nerve endings and the number of osteoclasts in subchondral bone also increased sharply in the Si*Alix*-Sr-SMSC-EXOs group compared to the Sr-SMSC-EXOs group (Fig. 4f, g, Fig. S5e). These data demonstrate the critical role of Alix in the enhanced pain relief offered by Sr-SMSC-EXOs.

### Central role of miR-143-3p in Sr-SMSC-EXOs in enhancing TMJOA alleviation

**Fig. 5:**
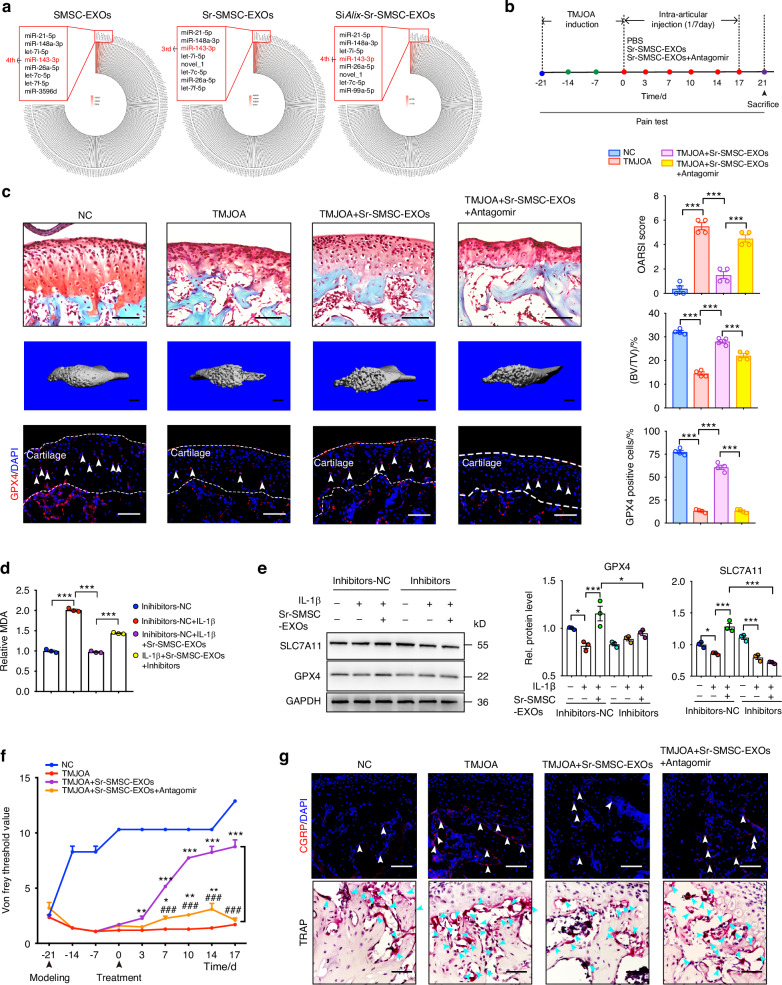
Central role of miR-143-3p in Sr-SMSC-EXOs in enhancing TMJOA alleviation. **a** Circular heatmap analysis of miR-143-3p expression abundance in the SMSC-EXOs, Sr-SMSC-EXOs, and Si*Alix*-Sr-SMSC-EXOs. **b** Schematic model of the time course for establishment of unilateral anterior crossbite (UAC) model of TMJOA mice with indicated treatment and pain test time. **c** Safranin-O/Fast green staining, OARSI scoring system, and immunofluorescence staining for GPX4 in condylar cartilage, and 3D reconstruction of the condyles and BV/TV in subchondral bone post indicated treatment. Arrow heads indicated positive cells. Scale bar for Safranin-O/Fast green staining and immunofluorescence staining, 100 μm; Scale bar for 3D reconstruction, 1 mm. **d** Relative MDA measurement in condylar chondrocytes 48 h after indicated treatment. **e** Western blot analyses of the GPX4 and SLC7A11 in condylar chondrocytes 48 h after indicated treatment. **f** Measurement of the pain threshold value in TMJ region by Von Frey monofilaments testing. Compared to TMJOA group, *P < 0.05, **P < 0.01, ***P < 0.001; compared to TMJOA+Sr-SMSC-EXOs group, ^###^*P* < 0.001. **g** Immunofluorescence staining for CGRP and TRAP staining in subchondral bone. Scale bar, 100 μm. Arrowheads indicate positive cells. Data are represented as mean ± SEM. *P < 0.05, ***P < 0.001

According to the analysis of the top 200 significantly expressed miRNAs in each type of EXOs, miR-143-3p, which ranked third in overall miRNAs of Sr-SMSC-EXOs (Fig. 5a), was hypothesized to play a pivotal role due to its high abundance and closely association with Sr and Alix. Here, we administered an antagonist of miR-143-3p, known as Antagomir-143-3p, to TMJOA mice through intra-articular injection (Fig. 5b). Histological and imaging assays showed that cartilage degradation was exacerbated, accompanied by drastically reduced BV/TV and Tb. Th in the TMJOA group treated with Sr-SMSC-EXOs+Antagomir compared to those in the TMJOA+Sr-SMSC-EXOs group (Fig. 5c, Fig. S6a). Furthermore, ferroptosis related GPX4^+^ chondrocytes also significantly decreased in the TMJOA+Sr-SMSC-EXOs+Antagomir group (Fig. 5c). In vitro, the downregulated cellular MDA level and upregulated expression of GPX4 and SLC7A11 in IL-1β-treated chondrocytes after Sr-SMSC-EXOs administration was markedly reversed by miR-143-3p inhibitor (Fig. 5d, e). Von Frey testing showed that the pain threshold appeared to decrease at the beginning of Antagomir-143-3p injection in TMJOA mice treated with Sr-SMSC-EXOs. The differences were significant at 7 days post-first injection and peaked at about a multiple of 4.15 at 17 days post-injection (*P* < 0.001) (Fig. 5f). Consistently, the density of CGRP^+^ nerve endings and osteoclast activity increased significantly with Antagomir-143-3p injection (Fig. 5g, Fig. S6b). Collectively, these findings identify miR-143-3p as a central beneficial miRNA in Sr-SMSC-EXOs for arthritis alleviation and further confirm the role of Sr and Alix in exosomal miRNA loading and TMJOA remission.

### MiR-143-3p targets Mfsd8 to reduce the ferroptosis susceptibility of chondrocytes in TMJOA attenuating

**Fig. 6:**
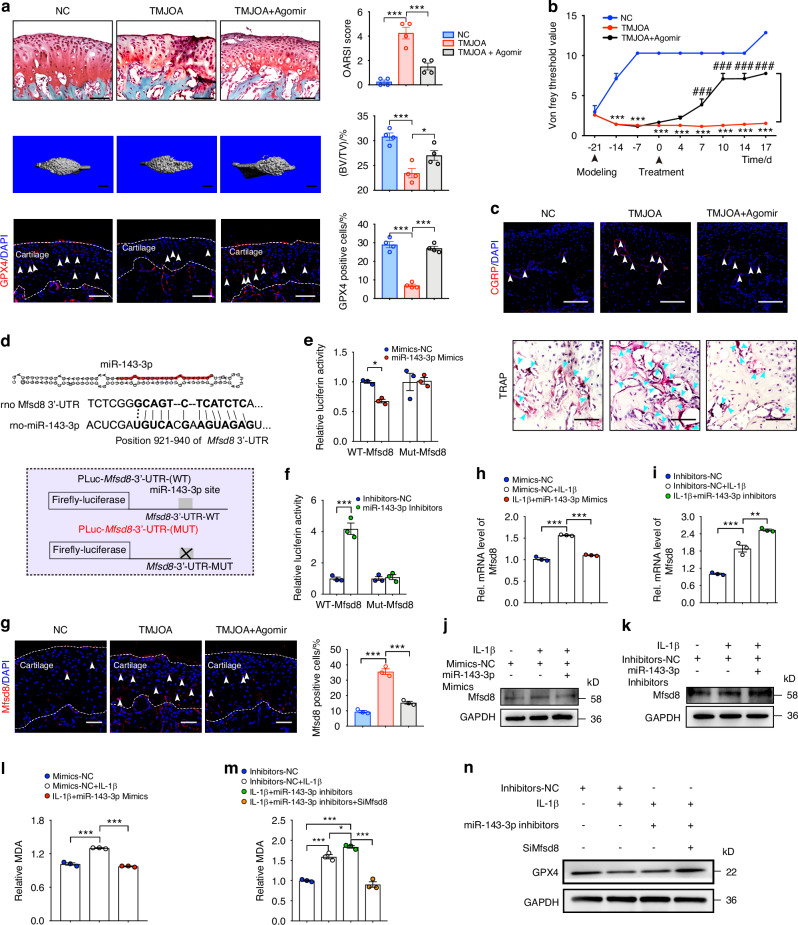
MiR-143-3p targets Mfsd8 to reduce the ferroptosis susceptibility of chondrocytes in TMJOA attenuating. **a** Safranin-O/Fast green staining, OARSI scoring system, and immunofluorescence staining for GPX4 in condylar cartilage, and 3D reconstruction of the condyles and BV/TV in subchondral bone. Arrowheads indicate positive cells. Scale bar for Safranin-O/Fast green staining and immunofluorescence staining, 100 μm; Scale bar for 3D reconstruction, 1 mm. **b** Measurement of the pain threshold value in TMJ region by Von Frey monofilaments testing. Compared to Sham group, ***P < 0.001; compared to TMJOA group, ^###^*P* < 0.001. **c** Immunofluorescence staining for CGRP and TRAP staining in subchondral bone. Scale bar, 100 μm. Arrowheads indicate positive cells. **d** The structure of miR-143-3p and the miR-143-3p sequence and the predicted miR-143-3p target site in the 3′-UTR of Mfsd8 (upper). Schematic of the luciferase reporter plasmids with the WT or MUT Mfsd8 3′-UTRs (lower). **e**, **f** Relative luciferase activity in HEK293T cells transfected with the indicated luciferase reporter plasmids along with miR-143-3p mimics or inhibitors, respectively. **g** Immunofluorescence staining for Mfsd8 in condylar cartilage. Arrowheads indicate positive cells. Scale bar, 100 μm. **h**, **i** Quantitative RT-PCR and **j**, **k** western blot analyses of the Mfsd8 in condylar chondrocytes 48 h after indicated treatment. **l**, **m** Relative MDA measurement in condylar chondrocytes 48 h after indicated treatment. **n** Western blot analyses of the GPX4 in condylar chondrocytes 48 h after indicated treatment. Data are represented as mean ± SEM. *P < 0.05, ***P < 0.001

To further clarify the role of miR-143-3p in TMJOA, we administered Agomir-143-3p to TMJOA mice. Safranin-O/Fast green staining and OARSI scores showed that cartilage degeneration was attenuated by Agomir-143-3p administration (Fig. 6a). Furthermore, the reduced BV/TV and GPX4^+^ chondrocytes as well as the increased Tb. Sp in TMJOA mice were rescued by Agomir-143-3p injection (Fig. 6a, and Fig. S7a). The pain threshold elevated sharply after Agomir-143-3p administration and reached a peak at 10 days post-first injection (Fig. 6b). Moreover, Agomir-143-3p significantly downregulated the CGRP expression and osteoclast activity of subchondral bone in TMJOA (Fig. 6c, Fig. S7b). These data imply that Agomir-143-3p alleviated TMJOA progression mainly by suppressing cartilage degeneration and osteoclast-induced joint pain.

Next, miRDR, MiRWalk 2.0, and TargetScan were used to predict target genes of miR-143-3p, which showed that the 3′-untranslated region (UTR) of the Mfsd8 mRNA might be the target (Fig. 6d). A further dual-luciferase reporter assay observed that miR-143-3p mimics retarded the activity of the wild-type (WT) reporter but not that of the mutant (MUT) reporter (Fig. 6e). Conversely, miR-143-3p inhibitor promoted the WT reporter activity but not that of the MUT reporter (Fig. 6f). Moreover, miR-143-3p mimics decreased the levels of Mfsd8 in primary chondrocytes, while miR-143-3p inhibition increased them (Fig. S7c, d). Immunostaining showed that the quantity of Mfsd8^+^ chondrocytes in TMJOA mice as about 3.75 times that in control mice. Agomir-143-3p injection improved the pathological increase of Mfsd8^+^ chondrocytes (Fig. 6g). Consistently, a decrease of Mfsd8^+^ chondrocytes was observed in Sr-SMSC-EXOs treated TMJOA mice whereas Antagomir-143-3p administration increased Mfsd8 (Fig. S7e). In addition, MiR-143-3p mimics hampered Mfsd8 and MDA upregulation induced by IL-1β, while miR-143-3p inhibitors had the opposite effect in vitro (Fig. 6h-m). These results demonstrate that miR-143-3p targets Mfsd8 in an inhibitory manner.

Then, we performed further experiments to clarify whether miR-143-3p protects chondrocytes from ferroptosis by directly targeting Mfsd8. First, we generated *Mfsd8* RNAi (Si*Mfsd8*) and verified that, compared to the group treated with IL-1β+miR-143-3p Inhibitors, the level of MDA in the group treated with IL-1β+miR-143-3p Inhibitors+Si*Mfsd8* significantly decreased (Fig. 6m). Moreover, the suppressive effect of the miR-143-3p inhibitors on the expression of GPX4 was reversed in *Mfsd8* RNAi-transfected cells (Fig. 6n, Fig. S7f). These data indicate that miR-143-3p reduced the susceptibility of chondrocytes to ferroptosis by targeting the Mfsd8 3′-UTR.

### Synovium niche-derived MSCs, but not bone marrow-derived MSCs, undergo Alix-mediated exosomal miRNA loading upon Sr exposure

**Fig. 7:**
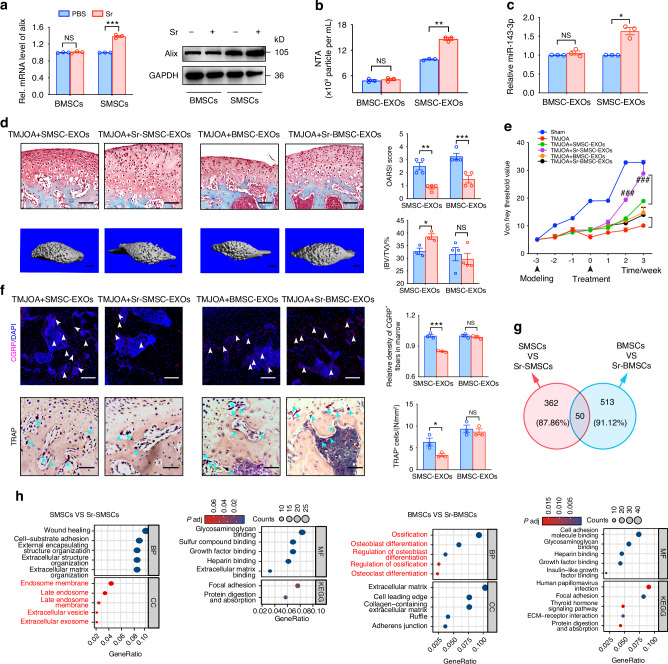
Synovium niche-derived MSCs, but not bone marrow-derived MSCs, undergo Alix-mediated exosomal miRNA loading upon Sr exposure. **a** Quantitative RT-PCR and western blot analyses of the Alix in MSCs 48 h after indicated treatment. **b** Number of particles was assessed by nanoparticle tracking analysis. **c** Quantitative RT-PCR analyses of the miR-143-3p in the SMSC-EXOs, Sr-SMSC-EXOs, BMSC-EXOs, and Sr-BMSC-EXOs. **d** Safranin-O/Fast green staining, OARSI scoring system in condylar cartilage, and 3D reconstruction of the condyles and BV/TV in subchondral bone post indicated treatment. Scale bar for Safranin-O/Fast green staining, 100 μm; Scale bar for 3D reconstruction, 1 mm. **e** Measurement of the pain threshold value in TMJ region by Von Frey monofilaments testing. Compared to Sham group, ***P < 0.001; compared to TMJOA + SMSC-EXOs group, ^###^*P* < 0.001. **f** Immunofluorescence staining for CGRP and TRAP staining in subchondral bone. Scale bar, 100 μm. Arrowheads indicate positive cells. **g** Venn diagram of the differently expressed mRNAs between BMSCs and Sr-BMSCs, and between SMSCs and Sr-SMSCs. **h** GO and KEGG pathway analyses of the target genes of the differently expressed mRNAs between SMSCs and Sr-SMSCs, and between BMSCs and Sr-BMSCs. Data are represented as mean ± SEM. *P < 0.05, ***P < 0.001. NS, not significant

Previous studies have addressed the pivotal functions of the site-specific stromal niche cells in pathologic and physiologic circumstances.^53,54^ SMSCs and BMSCs are known to be important sources of active miRNA and EXOs in osteoarthritis. To further investigate whether the Sr/Alix strategy for enhancing the therapeutic efficacy of MSC-EXOs against OA is specifically applicable only for the synovial stromal niche cells, we first compared the changes of Alix in SMSCs and BMSCs after Sr exposure. Interestingly, the mRNA and protein levels of Alix in BMSCs were not altered upon Sr exposure (Fig. 7a). In addition, neither the number of EXOs nor the level of miR-143-3p in Sr-BMSC-EXOs differed from those of BMSC-EXOs (Fig. 7b, c). However, SMSCs responded to Sr exposure with increases in the levels of Alix, EXOs and miR-143-3p. These findings suggest that synovium-derived MSCs, but not bone marrow-derived MSCs, undergo exosomal miRNA loading via Alix upon Sr exposure.

Next, we compared the in vivo therapeutic effects of EXOs derived from SMSCs and BMSCs, with and without Sr pretreatment, in TMJOA mice. Safranin-O/Fast green staining showed that Sr-BMSC-EXOs reduce the OARSI score more than BMSC-EXOs in TMJOA mice (Fig. 7d, Fig. S8a). However, unlike the group with SMSCs, there was no prominent improvement of BV/TV or Tb. Sp in the Sr-BMSC-EXOs group compared to the BMSC-EXOs group (Fig. 7d, Fig. S8a). Throughout the experiments, no significant differences were manifested in pain threshold, density of CGRP^+^ fibers or osteoclasts activity between Sr-BMSC-EXOs and BMSC-EXOs injected into TMJOA mice (Fig. 7e, f, Fig. S8b). These data suggest that the effects of Sr on regulating exosome biogenesis vary with the source of the MSCs and confirm the importance of the synovium niche-derived MSCs in the strategy to increase Alix using Sr to enhance the effectiveness of MSC-EXOs in TMJOA.

To further analyze the different responses of SMSCs and BMSCs to Sr exposure, an mRNA sequencing assay was employed to explore the different changes of mRNA profiles in BMSCs and SMSCs after Sr exposure. The Venn diagram showed that there were only 50 overlapping differentially expressed (DE) genes. In the dataset comparing SMSCs with and without Sr pretreatment, 362 (87.86%) of the genes were entirely different, as were 513 (91.12%) of the genes in dataset comparing BMSCs with and without Sr pretreatment (Fig. 7g). Further GO and KEGG analysis showed that 412 DE genes in the SMSCs vs. Sr-SMSCs dataset were associated with exosome biogenesis and chondrocyte metabolism, such as ‘external encapsulating structure organization,’ ‘extracellular structure organization’ and ‘extracellular matrix organization’ biological processes, and ‘endosome membrane,’ ‘late endosome,’ ‘late endosome membrane,’ ‘extracellular vesicle’ and ‘extracellular exosome’. However, 563 DE genes in the BMSCs vs. Sr-BMSCs dataset were not involved in the above functions, but rather were associated with aspects of bone remodeling, such as ‘ossification,’ ‘osteoblast differentiation,’ ‘regulation of osteoblast differentiation,’ ‘regulation of ossification’ and ‘osteoclast differentiation’ biological processes, and ‘extracellular matrix,’ ‘cell leading edge,’ ‘collagen-containing extracellular matrix,’ ‘ruffle’ and ‘adherens junction’ (Fig. 7h). The above data further reveal the unique alterations of transcriptome profiles in SMSCs and BMSCs after Sr exposure.

## Discussion

**Fig. 8:**
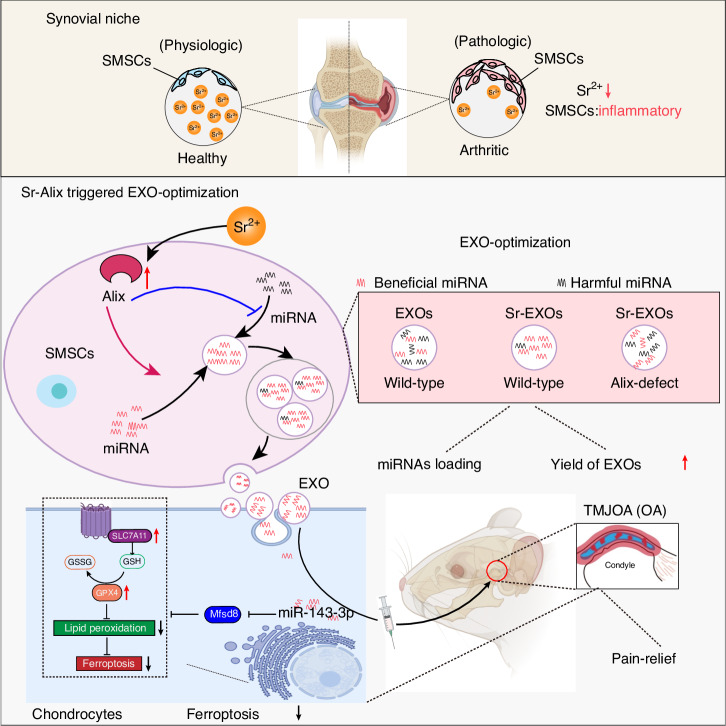
Schematic diagram shows a potential strategy, utilizing a niche cell-guided work pattern, to optimize the miRNA compositions in therapeutic SMSC-EXOs and boost the yield of the EXOs

The advantages of MSC-EXOs in OA therapy lie in their desirable characteristics, including low immunogenicity, nanoscale size, stable biological properties, and convenient storage and more. In this study, we revealed that the interaction between Sr and Alix augments the therapeutic potential of synovial mesenchymal stem cell-derived exosomes by refining their miRNA cargo (Fig. 8). This trace element-driven modification not only underscores the importance of cellular microenvironment in exosome biogenesis but also highlights a novel mechanism of site-specific miRNA loading.

Trace elements are essential for maintaining physiological functions and homeostasis. Aberrant level of elements, especially insufficient amounts, can lead to the occurrence of various diseases.^55^ Sr ion is especially critical for bone and joint health. Numerous studies have suggested Sr as an important effective component in some drugs and biomaterials for osteoporosis, arthritis, critical bone defect, endocrine diseases, and neuromuscular impediments.^13,30–36,56,57^ Previous studies have established Mg^2+^-mediated decrease in the expression of miR-381 in macrophage-derived exosomes as a novel strategy for the promotion of the osteogenic differentiation of BMSCs.^58^ Wang et al. proposed that copper could down-regulate anti-angiogenic RNAs and up-regulate the pro-angiogenic RNAs in EXOs.^25^ Li-bioactive glass promoted the angiogenic capacity of endothelial cells by enhancing the expressions of miR-130a in BMSCs-derived EXOs.^59^ In summary, previous studies like the ones noted here have focused on the influence of trace element on certain types of miRNAs, such as miR-381 and miR-130a, and explored their roles in disease progress and alleviation.^58,59^ However, the therapeutic efficacy of EXOs is jointly regulated by the overall profile of active exosomal miRNAs, including beneficial and harmful components. As far as we were aware, this is the first study deciphering the overall alterations of exosomal miRNAs elicited by a trace element using small RNA sequencing. Previous studies have demonstrated that miR-146a and miR-26a-5p are harmful and miR-140-3p is beneficial for OA recovery.^13,17,60–63^ In our study, the abundance of miR-146a and miR-26a-5p decreased and miR-140-3p increased in Sr-SMSCs-EXOs. Moreover, we also identified upregulated novel-261, novel-1(data not shown) and miR-143-3p as beneficial miRNA components for arthritis alleviation in vivo. Thus, Sr not only boosted the secretion of SMSC-EXOs, but more importantly, optimized the miRNA compositions in EXOs to include more beneficial and less harmful miRNAs partially via Alix. These results confirmed the role of Sr in controlling exosomal miRNA loading and partially revealed the Alix-mediated mechanism in arthritis. Notably, our data also revealed that synovium niche-derived MSCs, but not bone marrow-derived MSCs, undergo Alix-mediated exosomal miRNA sorting upon Sr exposure. Transcriptome analysis showed that the DE genes of SMSCs upon Sr exposure were involved in EXOs biogenesis and chondrocyte metabolism, but those of BMSCs upon Sr exposure were not, instead being involved in bone remodeling. The entirely different manners in which miR-143-3p responded to Sr in the present study might be attributed to the different source and type of the MSCs used. The above data thus provide a hint concerning the ‘niche cell-guided’ work pattern of Sr in controlling exosomal miRNA loading. There is still more work to be done to fully reveal the mechanism by which niche cells may guide the role of Sr in regulating exosome biogenesis.

In this study, we demonstrated that Alix is required for the Sr-triggered miRNA loading in SMSCs by conducting a detailed analysis of functionally known and unknown DE miRNAs. Knockdown of Alix reversed the role of Sr in promoting EXOs secretion and specific miRNA sorting, e.g., loading more ‘beneficial’ and less ‘harmful’ miRNAs into EXOs. To date, the mechanism by which Alix mediates the miRNA loading is largely unknown. The process of miRNA loading is associated with RNA-binding proteins such as Ago2 and hnRNPA2B1, as well as membranous proteins such as Cav-1 and neural sphingomyelinase 2.^64^ A recent study confirmed that YBX1 recruited and sorted miR-223 into EXOs, a process which was impeded by perturbing phase separation.^65^ There is also evidence that PDZ domains of syntenin directly bind with CD63 and syndecans19 to support exosomal sorting.^66^ Hsp90α is sorted into EXOs through the interaction with Rab coupling protein.^67^ Alix can nucleate ESCRT-III onto the MVBs limiting membrane and identify cargo through an in Alix-dependent mechanism.^68,69^ Whether Alix interacts with ESCRT and interferes specific with miRNA binding is worth further exploring.

MiR-143-3p is decreased in the blood and dorsal root ganglia of collagen-induced arthritic mice and in the articular cartilage of old OA lesioned samples, the inhibition of which may result in depletion of cellular components and lead to increased apoptosis by the activating of EIF2 signaling.^70,71^ Yang reported that miR-143-3p targets IGF1R and IGFBP5 to regulate the proliferation and apoptosis of synovial cell lines by the Ras/p38 MAPK signaling pathway in RA.^72^ In this study, we identified miR-143-3p as an important therapeutic miRNA in Sr-SMSC-EXOs and SMSC-EXOs. At first, we noticed miR-143-3p for its high abundance in the total miRNA and upregulated expression after Sr exposure. Then, blockage of miR-143-3p by AntagomiR-143-3p was shown to impede the therapeutic effects of Sr-SMSC-EXOs. Administration of Agomir-143-3p attenuated TMJOA. Furthermore, we found that the expression of miR-143-3p was regulated by Alix. Mfsd8 was predicted to act upstream of TORC1 signaling and participate in cellular catabolic processes.^73^ Yi et al. demonstrated that hyperactive mutation of PI3K-AKT-mTOR signaling prevents cancer cells from ferroptosis via SREBP1/SCD1-mediated lipogenesis.^74^ However, to date, whether miR-143-3p acts as upstream of Mfsd8 is not clear. We showed that Mfsd8 was obviously upregulated in TMJOA damaged cartilage, and this upregulation was reversed after AgomiR-143-3p treatment. Knockdown of Mfsd8 significantly reversed the enhancement of ferroptosis by miR-143-3p inhibitors. Moreover, dual-luciferase reporter assay observed that miR-143-3p mimics retarded the activity of the WT reporter but not that of the mutant (MUT) reporter, confirming that Mfsd8 is the direct target of miR-143-3p with a role in suppressing chondrocyte ferroptosis and ameliorating TMJOA. To our knowledge, this is the first study to report the biological significance of miR-143-3p in ameliorating TMJOA by directly targeting Mfsd8 to inhibit chondrocyte ferroptosis. It suggests that miR-143-3p could be a novel target for attenuating TMJOA.

There are several recognized limitations to our study. First, miRNAs are only one type of EXO cargo among many, such as lipids, proteins, and metabolites. To have a more comprehensive understanding of the influence of Sr exposure on SMSC-EXOs, more sequencing techniques and rigorous verification will be required to analyze different kinds of exosomal cargo, so that the alteration of EXOs can be explained more deeply. Second, in this study, we have demonstrated that SMSC-EXOs and Sr-SMSC-EXOs alleviate cartilage injury by inhibiting chondrocyte ferroptosis, and we observed that these EXOs inhibited abnormal remodeling of subchondral bone. However, we have not yet fully revealed the specific mechanism by which SMSC-EXOs and Sr-SMSC-EXOs affect subchondral bone, or whether there is crosstalk between cartilage and subchondral bone in this process. This will be the subject of future investigations. Third, there is a great unmet medical need for a disease-modifying biological reagent to combat OA. Although we have indicated the feasibility of using SMSCs EXOs to treat TMJOA, this is just the first step toward the translation of our research into clinical use. Future experiments should test the efficacy of SMSC-EXOs and Sr-SMSC-EXOs in TMJOA using large-animal models. Further investigation of appropriate EXO dosage and administration will promote the clinical application of EXOs.

In conclusion, our results demonstrate that treatment of SMSCs with Sr not only significantly enhanced the yield of SMSC-derived exosomes, but also optimized the miRNA profile within these exosomes. Functional assays indicated that Sr-SMSC-EXOs were more effective in attenuating chondrocyte ferroptosis and osteoclast-mediated joint pain, compared to untreated SMSC-EXOs. Furthermore, our data suggest a mechanistic underpinning for these effects, highlighting the involvement of the Alix protein in Sr-induced miRNA loading. These findings establish a direct correlation between Sr treatment, Alix-mediated miRNA selectively loading, site-specific SMSCs and therapeutic efficacy in the context of TMJOA.

## Materials and Methods

### Animals

Sprague Dawley rats (8 weeks old, male, weighing an average of 200 g) and C57BL/6 mice (8 weeks old, male, weighing an average of 20 g) were purchased from Dossy Experimental Animal Limited Company (Chengdu, China). All the animals were bred and maintained under specific-pathogen-free (SPF) conditions in a 12-/12-h light/dark cycle, with free access to food and water. Animals were anaesthetized with pentobarbital sodium (100 mg/kg, injected intraperitoneally). A previously established unilateral anterior crossbite (UAC) model was used to induce TMJOA. Two metal tubes (Xinhua Pharmaceutical Co. LTD, Shandong, China) were respectively bonded to the left maxillary and mandibular incisor. From the next day, the instrument was checked every other day and re-bonded in a timely manner if it was displaced. Soft food was provided for the first three days after the operation and a standard diet was resumed from the fourth day. Animals were sacrificed according to the schematic diagram after different interventions for analyses. All procedures were approved by the Ethics Committee of the West China School of Stomatology, Sichuan University under document number WCHSIRB-D-2021-472.

To assess the effect of SMSC-EXOs and Sr-SMSC-EXOs on TMJOA, animals were randomly allocated to four groups: Sham, TMJOA, TMJOA + SMSC-EXOs, and TMJOA+Sr-SMSC-EXOs. Animals in the Sham and TMJOA group received equivalent PBS treatment. For experiments assessing the effects of SMSC-EXOs, Sr-SMSC-EXOs, and Si*Alix*-Sr-SMSC-EXOs on TMJOA, animals were randomly allocated to five groups: Sham, TMJOA, TMJOA + SMSC-EXOs, TMJOA+Sr-SMSC-EXOs, and TMJOA+Si*Alix*-Sr-SMSC-EXOs. Animals in Sham and TMJOA group received equivalent PBS treatment. Exosomes (25 μL EXOs suspension for every rat articular cavity) or equal volume of PBS were injected into the TMJ articular cavity. Upregulation or downregulation of miR-143-3p expression in animals was achieved by TMJ articular cavity injection of synthetic miR-143-3p agomir (250 nmol/kg body weight) or miR-143-3p antagomir (250 nmol/kg body weight), with miR-NC used as a negative control (250 nmol/kg body weight). Sequences are shown in Tab. S3. These modified miRNA agomir/antagomir exhibit high affinity to cell membranes and demonstrate increased stability and efficacy in vivo experiments. To assess the effect of miR-143-3p antagomir on TMJOA, animals were randomly allocated to four groups: negative control (NC), TMJOA, TMJOA+Sr-SMSC-EXOs, and TMJOA+Sr-SMSC-EXOs+miR-143-3p antagomir. Animals in the NC group, TMJOA group, and TMJOA+Sr-SMSC-EXOs groups were administered a miR-143-3p antagomir negative control. To assess the effect of miR-143-3p agomir on TMJOA, animals were randomly allocated to three groups: NC, TMJOA, TMJOA+agomir. Animals in the NC group and TMJOA group were applied with a miR-143-3p agomir negative control. For experiments assessing the effect of SMSC-EXOs, Sr-SMSC-EXOs, BMSC-EXOs, Sr-BMSC-EXOs on TMJOA, animals were randomly allocated to six groups: Sham, TMJOA, TMJOA + SMSC-EXOs, and TMJOA+Sr-SMSC-EXOs, TMJOA + BMSC-EXOs, and TMJOA+Sr-BMSC-EXOs. Exosomes (10 μL EXOs suspension for every mouse articular cavity) or an equal volume of PBS were injected into the TMJ articular cavity.

### Isolation and identification of SMSCs

Synovial mesenchymal stem cells (SMSCs) were isolated from synovial tissue of 6-week-old SD rats. The cells were routinely cultured in high glucose DMEM medium (Gibco, USA) supplemented with 10% fetal bovine serum (FBS; BioInd, Kibbutz, Israel) and 1% penicillin-streptomycin (Gibco, USA) at 37 °C in a humidified incubator with 5% CO_2_ and 95% humidity. To further isolate and purify stem cells, single cell suspensions of primary cells were cloned using the limiting dilution method. SMSCs from the same passage (passages 3-6) were used in each experiment. For identification of SMSCs, cells were analyzed for the expression of cell surface markers by flow cytometric analysis. For determining the multipotential differentiation capabilities of SMSCs, cells were cultured in osteogenesis-inducing medium, adipogenesis-inducing medium, and chondrogenesis-inducing medium for 21 days. They were then fixed and stained with ALP for osteocytes, Oil Red O for adipocytes, and Toluidine blue and Safranin-O for pellet culture chondrocytes.

### Preparation and characterization of SMSCs exosomes

SMSCs were cultured in high-glucose DMEM containing 10% exosome-free FBS (Gibco, USA) or in high-glucose DMEM containing 10% exosome-free FBS and 100 nmol/L trontium chloride, in an equal volume of PBS for 48 h to collect conditioned medium (CM). Exosomes were separated, extracted, and purified from CM as previously described. Exosomes were resuspended with 100 μL PBS and stored in a -80°C freezer until use. The morphology of exosomes was observed under TEM (HITACHI H-7000FA, Japan). The particle size distribution of exosomes was analyzed by NTA (Malvern, UK). Western blot was used to identify the protein expression levels.

### Histology and immunofluorescence

After animals were sacrificed, bilateral TMJs were separated, fixed with 4% paraformaldehyde solution for 24 h and stored in 70% ethanol at 4 °C before further processing TMJs were decalcified using 0.5 mol/L EDTA for 2 months, dehydrated, embedded in paraffin, and sectioned to 5-μm thickness used for staining. According to the manufacturers’ recommendations, the sections were stained with Safranin-O/Fast green (Solarbio, Beijing, China) and TRAP staining (Sigma-Aldrich, USA). The morphology of the articular cartilage was observed using a microscope, and the severity of the TMJOA-like phenotype was evaluated using the OARSI scoring system. For immunofluorescence analysis, after antigen retrieval and blocking, frozen sections or fixed chondrocytes were incubated at 4 °C overnight with primary antibodies against MMP13 (Abcam, ab39012, 1:100), GPX4 (Abcam, ab125066, 1:100), CGRP (CST, 14959, 1:100), or Mfsd8 (ProteinTech, 24298-1-AP, 1:100). The secondary antibodies were used donkey anti-mouse Alexa Fluor 488/555/647 and donkey anti-rabbit Alexa Fluor 488/555/647 (Thermo Fisher Scientific). The nuclei were counterstained with DAPI (Sigma-Aldrich, D9542), the images of the samples were observed through a Nikon A1 confocal microscope, processed, and analyzed with ImageJ software. Sections were blinded and scored by two experienced researchers, and the average scores were used in statistical analyses.

### Von Frey monofilaments testing

The Von Frey monofilaments testing (Stoelting, 58011) is often used to measure pain experienced by animals. The tip of a hard plastic filament was used to stimulate the test area of the animal, and the animal’s reaction was observed to judge whether it evidenced a painful response to the stimulus. The animals were kept calm before the pain threshold detection. The midpoint of the line between the corner of the eye and the ear was used as the test area (‘temporomandibular zone’). The intensity of the stimulus (g) was recorded when the animal was observed to undergo a change from a calm state to a mouth-rubbing or grasping response. Starting from the minimal filament intensity of the minimum filaments, the test was repeated for three times at an interval of 30 seconds. The average value was taken as the pain threshold of the temporomandibular zone.

### Micro-computed tomography

Micro-CT scanning of TMJs was performed on μCT 80 system (filter Al 0.2 mm, 70 kV, 200 µA, 10.0 μm, SCANCO Medical AG, Switzerland) with a resolution of 10 μm to detect the changes in the subchondral bone. Three-dimensional images were reconstructed by SCANCO Visualizer for morphological assessment. Bone density was analyzed by SCANCO Evaluation, including the ratio of bone volume to tissue volume (BV/TV), trabecular space (Tb. Sp), and trabecular thickness (Tb. Th).

### Isolation and culture of TMJ condylar chondrocytes

TMJ condylar cartilage was dissected from 2-week-old SD rats. Briefly, tissues were washed 3 times with PBS, dissected into pieces, and then digested with 2.5 mg/mL collagenase type II (Gibco) for 2 h and 0.5 mg/mL collagenase type II overnight at 37 °C. The primary condylar chondrocytes were resuspended and cultured in low glucose DMEM medium (Gibco) containing 10% FBS and 1% penicillin-streptomycin at 37 °C in a humidified incubator with 5% CO_2_ and 95% humidity. To guarantee the phenotype integrity, we only use first-passage chondrocytes were used for experiments employing the in vitro chondrocyte model of OA. Primary condylar chondrocytes were incubated with recombinant IL-1β (10 ng/mL, Peprotech, 211-11B), SMSC-EXOs (5 μL/mL), Sr-SMSC-EXOs (5 μL/mL), Si*Alx*-Sr-SMSC-EXOs (5 μL/mL), and miR-143-3p mimics (50 nmol/L), miR-143-3p inhibitors (50 nmol/L), mimics NC (50 nmol/L) or inhibitors NC (50 nmol/L).

### CCK-8 cell viability assay and EDU assay

SMSCs were plated in 96-well plates at 3 500 cells per well in 100 μL of culture medium supplemented with 10 μL of CCK-8 reagent (MCE) and incubated at 37 °C for 2.5 h following indicated treatments. The absorbance was measured at 450 nm using a microplate reader (Synergy H1; BioTek). SMSCs labeled with the EDU working fluid for 2.5 h, and stained with DAPI. The images were acquired with fluorescence microscopy.

### MDA and GSH assay

After the treatments, the malonaldehyde (MDA) level and glutathione (GSH) level of primary condylar chondrocytes were determined with a Lipid Peroxidation MDA Kit (Beyotime, S0131) and GSH and GSSG Assay Kit (Beyotime, S0053) according to the manufacturers’ protocols.

### Lipid peroxidation and lipid droplet staining

After the treatments, primary condylar chondrocytes were incubated with 5 µmol/L of BODIPY581/591 C11 (Invitrogen, D3861) for 30 min, trypsinized and filtered into single cell suspensions. Flow cytometry analysis (Becton Dickinson) was performed using the FITC filter for oxidized BODIPY-C11 (emission: 510 nm) and PE-TexasRed filter for reduced BODIPY-C11 (emission: 590 nm). About 20 000 cells were analyzed for each sample. FlowJo v10 (BD Bioscience) was used for data analysis. After the treatments, primary condylar chondrocytes were stained with DCFH-DA (Beyotime, S0033) to measure ROS and assayed by fluorescence microscopy (Ts2R/FL; Nikon). Fixed chondrocytes were stained with 0.1 µg/mL Nile Red (MCE, HY-D0718) for 30 minutes, and the nuclei were counterstained using DAPI (Sigma). Images were captured with a Nikon A1 confocal microscope and analyzed with ImageJ software.

### Measurement of mRNA and miRNA

Tissue/cell total RNA was extracted from cultured cells using an Animal Total RNA Isolation Kit/Cell Total RNA Isolation Kit (Foregene, RE-03011/03111) according to the manufacturers’ instructions. For mRNA, the first-strand cDNA was prepared using HiScript II Q RT SuperMix for qPCR (Vazyme Biotech co., China). qRT-PCR was performed using SYBR Premix Ex Taq II (Vazyme Biotech co., China) in a CFX96 Real-Time System (Bio-Rad). GAPDH RNA was used as a housekeeping control. For miRNA, 1 μg total RNA was reverse transcribed using the All-in-One miRNA qRT-PCR Detection Kit (GeneCopoeia, China) according to the manufacturer instructions. U6 RNA was used as a housekeeping control. The primer sequences are listed in Tab. S4.

### Western blot

Primary condylar chondrocytes or exosomes lysates were extracted using RIPA lysis buffer (Beyotime, P0013B) containing PMSF (Beyotime, ST505). The protein concentration of the samples was detected using a BCA protein assay kit (Beyotime, #P0012). The samples were heated at 100 °C for 5 min in sample buffer, separated on 10% SDS-polyacrylamide gels, and transferred to PVDF membranes (Bio-Rad). The membranes were blotted with 5% BSA and incubated with primary antibody at 4 °C overnight. The membranes were washed in TBST solution and incubated with the secondary antibodies. The antibody-antigen complexes were visualized with Immobilon reagents (Millipore, WBKLS0100). The following primary antibodies were applied: GPX4 (Abcam, ab125066, 1:1 000), SLC7A11 (Abcam, ab175186, 1:1 000), Mfsd8 (ProteinTech, 24298-1-AP, 1:1 000), CD9 (Abclonal, A1703, 1:1 000), CD63 (Abclonal, A5271, 1:1 000), CD81 (Abclonal, A4863, 1:1 000), TSG101 (Abclonal, A5789, 1:1 000), Alix (Abclonal, A2215, 1:1 000), GAPDH (ProteinTech, 10494-1-AP, 1:5 000) and β-actin (ProteinTech, 66009-1-IG, 1:5 000)

### RNA interference (RNAi)

siRNAs specific to Alix were designed with the coding sequences of Alix shown in Tab. S5. SMSCs were seeded into 6-wells plates and transfected with siRNA (100 nmol/L using Lipofectamine 3000 (Invitrogen). Non-silencing siRNA was used as a negative control.

### Sequencing and data analysis

Small-RNA sequencing and miRNA data analysis of the SMSC-EXOs, Sr-SMSC-EXOs, and Si-Sr-SMSC-EXOs groups were conducted by Novogene (Beijing, China). Small-RNA sequencing was performed in triplicate using three independent sets of RNA preparations. DESeq analysis was used to identify differentially expressed miRNAs with a threshold of fold change ≥1.5 and *P* < 0.05. mRNA sequencing and mRNA data analysis of the BMSCs, Sr-BMSCs, SMSCs, and Sr-SMSCs groups were conducted by Novogene (Beijing, China). mRNA sequencing was performed in triplicate using three independent sets of RNA preparations. DESeq analysis was used to identify differentially expressed mRNAs with a threshold of fold change ≥1.5 and *P* < 0.05. The consistency rate was defined as sequencing screened miRNAs with the same trend of change as reported in literature/sequencing screened miRNAs with known functions in literature. The inconsistency rate was defined as sequencing screened miRNAs with the opposite trend of change as reported in literature/ sequencing screened miRNAs with known functions in literature.

### Dual-luciferase reporter assay

HEK293 cells (Shanghai Institutes for Biological Sciences of the Chinese Academy of Sciences, China) were seeded into 24-well plates and transfected upon achieving 80% confluence. A corresponding Mfsd8-luciferase reporter (pZX-FR02-MFSD8 3′-UTR) containing miR-143-3p-binding sites was co-transfected into HEK293 cells, respectively, with a mimic or inhibitor of miR-143-3p, or miR-NC, using the Lipofectamine 3000 (Invitrogen). Sequences are shown in Tab. S6. The cells were harvested at 48 h after transfection, the cells lysates were extracted, and luciferase activities of different samples were measured using a dual-luciferase reporter assay (Promega, Madison, WI, USA). The results were shown with relative luciferase activity (Firefly Luc/Renilla Luc).

### Isolation of BMSCs and BMSCs exosomes

Bone marrow mesenchymal stem cells (BMSCs) were isolated from bone marrow tissue of 2-week-old mice. The cells were routinely cultured in α-MEM medium (Gibco) supplemented with 10% fetal bovine serum (FBS; BioInd, Kibbutz, Israel) and 1% penicillin-streptomycin at 37 °C in a humidified incubator with 5% CO_2_ and 95% humidity. To further isolate and purify stem cells, single cell suspensions of primary cells were cloned with the limiting dilution method as previously described. BMSCs from the same passage (passages 3-6) were used in each experiment. BMSCs were cultured in high-glucose DMEM containing 10% exosome-free FBS (Gibco, USA) or in α-MEM medium containing 10% exosome-free FBS and 100 nmol/L strontium chloride or equal volume of PBS for 48 h to collect conditioned medium (CM). Exosomes were separated, extracted, and purified from CM as previously described. Exosomes were resuspended with 100 μL PBS and stored in -80 °C freezer until use.

### Statistical analysis

Data are presented as the mean ± standard error of at least three independent experiments. Significance of differences between two treatment groups was evaluated using Student’s t test. A Paired t test was used to determine the significance between the baseline and an endpoint within a group. For multiple comparisons of continuous measures between groups, when the variables were distributed normally, one-way analysis of variance (ANOVA) was performed, and Tukey’s post hoc test was applied to determine the statistical significance between groups. When the variables were not distributed normally, the Kruskal-Wallis’s test was used. All statistical analyses were conducted using GraphPad Prism 8. *P* < 0.05 was considered statistically significant.

## Supplementary information

Supplementary Materials

## Data Availability

The data used and/or analyzed during the current study are contained within the manuscript. Other data are available from the corresponding author on reasonable request.

## References

[1] Cao, H. et al. Cell-free osteoarthritis treatment with sustained-release of chondrocyte-targeting exosomes from umbilical cord-derived mesenchymal stem cells to rejuvenate aging chondrocytes. *ACS Nano* **17**, 13358–13376 (2023). 37439514 10.1021/acsnano.3c01612

[2] Hanai, H. et al. Small extracellular vesicles derived from human adipose-derived mesenchymal stromal cells cultured in a new chemically-defined contaminate-free media exhibit enhanced biological and therapeutic effects on human chondrocytes in vitro and in a mouse osteoarthritis model. *J. Extracell. Vesicles* **12**, e12337 (2023). 37367299 10.1002/jev2.12337 PMC10295161

[3] Kong, R., Ji, L., Pang, Y., Zhao, D. & Gao, J. Exosomes from osteoarthritic fibroblast-like synoviocytes promote cartilage ferroptosis and damage via delivering microRNA-19b-3p to target SLC7A11 in osteoarthritis. *Front Immunol.* **14**, 1181156 (2023). 37691947 10.3389/fimmu.2023.1181156 PMC10484587

[4] Xu, X. et al. Exosome-mediated delivery of kartogenin for chondrogenesis of synovial fluid-derived mesenchymal stem cells and cartilage regeneration. *Biomaterials* **269**, 120539 (2021). 33243424 10.1016/j.biomaterials.2020.120539

[5] You, D. G. et al. Metabolically engineered stem cell-derived exosomes to regulate macrophage heterogeneity in rheumatoid arthritis. *Sci. Adv.* **7**10.1126/sciadv.abe0083 (2021). 10.1126/sciadv.abe0083 PMC8172131 34078596

[6] Zhang, S. et al. MSC exosomes alleviate temporomandibular joint osteoarthritis by attenuating inflammation and restoring matrix homeostasis. *Biomaterials* **200**, 35–47 (2019). 30771585 10.1016/j.biomaterials.2019.02.006

[7] Bi, R. et al. Divergent chondro/osteogenic transduction laws of fibrocartilage stem cell drive temporomandibular joint osteoarthritis in growing mice. *Int J. Oral. Sci.* **15**, 36 (2023). 37626033 10.1038/s41368-023-00240-5 PMC10457315

[8] Tao, S.-C. et al. Exosomes derived from miR-140-5p-overexpressing human synovial mesenchymal stem cells enhance cartilage tissue regeneration and prevent osteoarthritis of the knee in a rat model. *Theranostics* **7**, 180–195 (2017). 28042326 10.7150/thno.17133 PMC5196895

[9] Zhu, Y. et al. Comparison of exosomes secreted by induced pluripotent stem cell-derived mesenchymal stem cells and synovial membrane-derived mesenchymal stem cells for the treatment of osteoarthritis. *Stem Cell Res Ther.* **8**, 64 (2017). 28279188 10.1186/s13287-017-0510-9 PMC5345222

[10] Zhang, B. et al. MiR-671 ameliorates the progression of osteoarthritis in vitro and in vivo. *Pathol. Res. Pr.* **215**, 152423 (2019). 10.1016/j.prp.2019.04.015 31085006

[11] Cao, Y. et al. Decreased miR-214-3p activates NF-κB pathway and aggravates osteoarthritis progression. *EBioMedicine* **65**, 103283 (2021). 33714889 10.1016/j.ebiom.2021.103283 PMC7957119

[12] Ni, Z. et al. The exosome-like vesicles from osteoarthritic chondrocyte enhanced mature IL-1β production of macrophages and aggravated synovitis in osteoarthritis. *Cell Death Dis.* **10**, 522 (2019). 31285423 10.1038/s41419-019-1739-2 PMC6614358

[13] Liu, L. et al. Bone marrow stromal cells stimulated by strontium-substituted calcium silicate ceramics: release of exosomal miR-146a regulates osteogenesis and angiogenesis. *Acta Biomaterialia* **119**, 444–457 (2021). 33129987 10.1016/j.actbio.2020.10.038

[14] Lee, E. S. et al. Reactive oxygen species-responsive dendritic cell-derived exosomes for rheumatoid arthritis. *Acta Biomaterialia* **128**, 462–473 (2021). 33878476 10.1016/j.actbio.2021.04.026

[15] Wu, Y. et al. Exosomes rewire the cartilage microenvironment in osteoarthritis: from intercellular communication to therapeutic strategies. *Int J. Oral. Sci.* **14**, 40 (2022). 35927232 10.1038/s41368-022-00187-z PMC9352673

[16] Liang, Y. et al. Chondrocyte-targeted microRNA delivery by engineered exosomes toward a cell-free osteoarthritis therapy. *ACS Appl. Mater. Interfaces* **12**, 36938–36947 (2020). 32814390 10.1021/acsami.0c10458

[17] Qin, H. et al. Silencing miR-146a-5p protects against injury-induced osteoarthritis in mice. *Biomolecules* **13**10.3390/biom13010123 (2023). 10.3390/biom13010123 PMC9856058 36671508

[18] Baloun, J. et al. Circulating miRNAs in hand osteoarthritis. *Osteoarthr. Cartil.* **31**, 228–237 (2023). 10.1016/j.joca.2022.10.021 36379393

[19] Wei, K. et al. Notch signalling drives synovial fibroblast identity and arthritis pathology. *Nature* **582**, 259–264 (2020). 32499639 10.1038/s41586-020-2222-z PMC7841716

[20] Croft, A. P. et al. Distinct fibroblast subsets drive inflammation and damage in arthritis. *Nature* **570**, 246–251 (2019). 31142839 10.1038/s41586-019-1263-7 PMC6690841

[21] Song, J. E. et al. Role of synovial exosomes in osteoclast differentiation in inflammatory arthritis. *Cells* **10**10.3390/cells10010120 (2021). 10.3390/cells10010120 PMC7827682 33435236

[22] Kato, T. et al. Exosomes from IL-1β stimulated synovial fibroblasts induce osteoarthritic changes in articular chondrocytes. *Arthritis Res Ther.* **16**, R163 (2014). 25092378 10.1186/ar4679 PMC4261911

[23] Zhang, Z. et al. Micro/nano-textured hierarchical titanium topography promotes exosome biogenesis and secretion to improve osseointegration. *J. Nanobiotechnology* **19**, 78 (2021). 33741002 10.1186/s12951-021-00826-3 PMC7980346

[24] Wu, Z., He, D. & Li, H. Bioglass enhances the production of exosomes and improves their capability of promoting vascularization. *Bioact. Mater.* **6**, 823–835 (2021). 33024902 10.1016/j.bioactmat.2020.09.011 PMC7530219

[25] Wang, Z. et al. Exosomes secreted by macrophages upon copper ion stimulation can promote angiogenesis. *Mater. Sci. Eng. C. Mater. Biol. Appl.* **123**, 111981 (2021). 33812609 10.1016/j.msec.2021.111981

[26] Abozaid, O. A. R. et al. Resveratrol-selenium nanoparticles alleviate neuroinflammation and neurotoxicity in a rat model of Alzheimer’s disease by regulating Sirt1/miRNA-134/GSK3β expression. *Biol. Trace Elem. Res.* **200**, 5104–5114 (2022). 35059981 10.1007/s12011-021-03073-7

[27] Othman, M. S., Hafez, M. M. & Abdel Moneim, A. E. The potential role of zinc oxide nanoparticles in microRNAs dysregulation in STZ-induced type 2 diabetes in rats. *Biol. Trace Elem. Res* **197**, 606–618 (2020). 31845207 10.1007/s12011-019-02012-x

[28] Tarale, P. et al. Manganese exposure: Linking down-regulation of miRNA-7 and miRNA-433 with α-synuclein overexpression and risk of idiopathic Parkinson’s disease. *Toxicol.* In Vitro **46**10.1016/j.tiv.2017.10.003 (2018). 10.1016/j.tiv.2017.10.003 28986288

[29] Niedermeier, W. & Griggs, J. H. Trace metal composition of synovial fluid and blood serum of patients with rheumatoid arthritis. *J. Chronic Dis.* **23**, 527–536 (1971). 5090325 10.1016/0021-9681(71)90128-7

[30] Reginster, J.-Y. et al. Efficacy and safety of strontium ranelate in the treatment of knee osteoarthritis: results of a double-blind, randomised placebo-controlled trial. *Ann. Rheum. Dis.* **72**, 179–186 (2013). 23117245 10.1136/annrheumdis-2012-202231 PMC3599139

[31] Yu, H. et al. Strontium ranelate promotes chondrogenesis through inhibition of the Wnt/β-catenin pathway. *Stem Cell Res. Ther.* **12**, 296 (2021). 34016181 10.1186/s13287-021-02372-z PMC8139050

[32] Bruyère, O. et al. Clinically meaningful effect of strontium ranelate on symptoms in knee osteoarthritis: a responder analysis. *Rheumatol. (Oxf.)* **53**, 1457–1464 (2014). 10.1093/rheumatology/keu018 24667161

[33] Meunier, P. J. et al. The effects of strontium ranelate on the risk of vertebral fracture in women with postmenopausal osteoporosis. *N. Engl. J. Med.* **350**, 459–468 (2004). 14749454 10.1056/NEJMoa022436

[34] Li, S. et al. Spontaneous immunomodulation and regulation of angiogenesis and osteogenesis by Sr/Cu-borosilicate glass (BSG) bone cement to repair critical bone defects. *Bioact. Mater.* **23**, 101–117 (2023). 36406252 10.1016/j.bioactmat.2022.10.021 PMC9664355

[35] Chiang, C.-W. et al. Strontium ranelate-laden near-infrared photothermal-inspired methylcellulose hydrogel for arthritis treatment. *Mater. Sci. Eng. C Mater. Biol. Appl* **123**, 111980 (2021). 33812608 10.1016/j.msec.2021.111980

[36] Wang, D. et al. Construction of Wogonin nanoparticle-containing strontium-doped nanoporous structure on titanium surface to promote osteoporosis fracture repair. *Adv. Health. Mater.* **11**, e2201405 (2022). 10.1002/adhm.202201405 36048734

[37] Ayyar, B. V. et al. CLIC and membrane wound repair pathways enable pandemic norovirus entry and infection. *Nat. Commun.* **14**, 1148 (2023). 36854760 10.1038/s41467-023-36398-z PMC9974061

[38] Laporte, M. H. et al. Alix is required for activity-dependent bulk endocytosis at brain synapses. *PLoS Biol.* **20**, e3001659 (2022). 35658004 10.1371/journal.pbio.3001659 PMC9200306

[39] Monypenny, J. et al. ALIX Regulates Tumor-Mediated Immunosuppression by Controlling EGFR Activity and PD-L1 Presentation. *Cell Rep.* **24**, 630–641 (2018). 30021161 10.1016/j.celrep.2018.06.066 PMC6077252

[40] Sun, R. et al. ALIX increases protein content and protective function of iPSC-derived exosomes. *J. Mol. Med (Berl.)* **97**, 829–844 (2019). 30944935 10.1007/s00109-019-01767-z

[41] Martin-Serrano, J. & Marsh, M. ALIX catches HIV. *Cell Host Microbe* **1**, 5–7 (2007). 18005675 10.1016/j.chom.2007.02.006

[42] Feng, Z. et al. A pathogenic picornavirus acquires an envelope by hijacking cellular membranes. *Nature* **496**, 367–371 (2013). 23542590 10.1038/nature12029 PMC3631468

[43] Lee, C.-P. et al. The ESCRT machinery is recruited by the viral BFRF1 protein to the nucleus-associated membrane for the maturation of Epstein-Barr Virus. *PLoS Pathog.* **8**, e1002904 (2012). 22969426 10.1371/journal.ppat.1002904 PMC3435242

[44] Zhang, M. et al. Identification of microRNA‑363‑3p as an essential regulator of chondrocyte apoptosis in osteoarthritis by targeting NRF1 through the p53‑signaling pathway. *Mol. Med Rep.* **21**, 1077–1088 (2020). 32016449 10.3892/mmr.2020.10940 PMC7003040

[45] Zhou, J.-L., Deng, S., Fang, H.-S., Peng, H. & Hu, Q.-J. CircSPI1_005 ameliorates osteoarthritis by sponging miR-370-3p to regulate the expression of MAP3K9. *Int Immunopharmacol.* **110**, 109064 (2022). 35978511 10.1016/j.intimp.2022.109064

[46] Zhou, X. et al. D-mannose alleviates osteoarthritis progression by inhibiting chondrocyte ferroptosis in a HIF-2α-dependent manner. *Cell Prolif.* **54**, e13134 (2021). 34561933 10.1111/cpr.13134 PMC8560605

[47] Zhu, S. et al. Subchondral bone osteoclasts induce sensory innervation and osteoarthritis pain. *J. Clin. Invest* **129**, 1076–1093 (2019). 30530994 10.1172/JCI121561 PMC6391093

[48] Larios, J., Mercier, V., Roux, A. & Gruenberg, J. ALIX- and ESCRT-III-dependent sorting of tetraspanins to exosomes. *J. Cell Biol.* **219**, e201904113 (2020). 32049272 10.1083/jcb.201904113 PMC7054990

[49] Ghossoub, R. et al. Syntenin-ALIX exosome biogenesis and budding into multivesicular bodies are controlled by ARF6 and PLD2. *Nat. Commun.* **5**, 3477 (2014). 24637612 10.1038/ncomms4477

[50] Ferreira, J. V. et al. LAMP2A regulates the loading of proteins into exosomes. *Sci. Adv.* **8**, eabm1140 (2022). 35333565 10.1126/sciadv.abm1140 PMC8956266

[51] Chen, C. et al. Tumor-suppressive circRHOBTB3 is excreted out of cells via exosome to sustain colorectal cancer cell fitness. *Mol. Cancer* **21**, 46 (2022). 35148775 10.1186/s12943-022-01511-1 PMC8832727

[52] Guan, L. et al. HRS phosphorylation drives immunosuppressive exosome secretion and restricts CD8+ T-cell infiltration into tumors. *Nat. Commun.* **13**, 4078 (2022). 35835783 10.1038/s41467-022-31713-6 PMC9283393

[53] Cambré, I. et al. Mechanical strain determines the site-specific localization of inflammation and tissue damage in arthritis. *Nat. Commun.* **9**, 4613 (2018). 30397205 10.1038/s41467-018-06933-4 PMC6218475

[54] Hernández-Camarero, P., López-Ruiz, E., Marchal, J. A. & Perán, M. Cancer: a mirrored room between tumor bulk and tumor microenvironment. *J. Exp. Clin. Cancer Res* **40**, 217 (2021). 34183054 10.1186/s13046-021-02022-5 PMC8240272

[55] Mertz, W. The essential trace elements. *Science* **213**, 1332–1338 (1981). 7022654 10.1126/science.7022654

[56] He, J.-L. et al. Associations of exposure to multiple trace elements with the risk of goiter: A case-control study. *Environ. Pollut.* **288**, 117739 (2021). 34245984 10.1016/j.envpol.2021.117739

[57] Miledi, R. Strontium as a substitute for calcium in the process of transmitter release at the neuromuscular junction. *Nature* **212**, 1233–1234 (1966). 21090447 10.1038/2121233a0

[58] Zhu, Y. et al. Mg2+ -mediated autophagy-dependent polarization of macrophages mediates the osteogenesis of bone marrow stromal stem cells by interfering with macrophage-derived exosomes containing miR-381. *J. Orthop. Res* **40**, 1563–1576 (2022). 34727384 10.1002/jor.25189

[59] Liu, L. et al. Lithium-containing biomaterials stimulate bone marrow stromal cell-derived exosomal miR-130a secretion to promote angiogenesis. *Biomaterials* **192**, 523–536 (2019). 30529871 10.1016/j.biomaterials.2018.11.007

[60] Yin, C. M. et al. Dysregulation of both miR-140-3p and miR-140-5p in synovial fluid correlate with osteoarthritis severity. *Bone Jt. Res* **6**, 612–618 (2017). 10.1302/2046-3758.611.BJR-2017-0090.R1 PMC5717073 29092816

[61] Al-Modawi, R. N., Brinchmann, J. E. & Karlsen, T. A. Multi-pathway protective effects of microRNAs on human chondrocytes in an in vitro model of osteoarthritis. *Mol. Ther. Nucleic Acids* **17**, 776–790 (2019). 31446120 10.1016/j.omtn.2019.07.011 PMC6716067

[62] Huang, Z. et al. MiR-26a-5p enhances cells proliferation, invasion, and apoptosis resistance of fibroblast-like synoviocytes in rheumatoid arthritis by regulating PTEN/PI3K/AKT pathway. *Biosci. Rep.* **39**10.1042/BSR20182192 (2019). 10.1042/BSR20182192 PMC6658817 31221815

[63] Ormseth, M. J. et al. Utility of Select Plasma MicroRNA for Disease and Cardiovascular Risk Assessment in Patients with Rheumatoid Arthritis. *J. Rheumatol.* **42**, 1746–1751 (2015). 26233505 10.3899/jrheum.150232 PMC4592411

[64] McKenzie, A. J. et al. KRAS-MEK Signaling Controls Ago2 Sorting into Exosomes. *Cell Rep.* **15**, 978–987 (2016). 27117408 10.1016/j.celrep.2016.03.085 PMC4857875

[65] Liu, X.-M., Ma, L. & Schekman, R. Selective sorting of microRNAs into exosomes by phase-separated YBX1 condensates. *Elife* **10**10.7554/eLife.71982 (2021). 10.7554/eLife.71982 PMC8612733 34766549

[66] Latysheva, N. et al. Syntenin-1 is a new component of tetraspanin-enriched microdomains: mechanisms and consequences of the interaction of syntenin-1 with CD63. *Mol. Cell Biol.* **26**, 7707–7718 (2006). 16908530 10.1128/MCB.00849-06 PMC1636879

[67] Zhang, S. et al. Mutant p53 Drives Cancer Metastasis via RCP-Mediated Hsp90α Secretion. *Cell Rep.* **32**, 107879 (2020). 32640214 10.1016/j.celrep.2020.107879

[68] Majer, O., Liu, B., Kreuk, L. S. M., Krogan, N. & Barton, G. M. UNC93B1 recruits syntenin-1 to dampen TLR7 signalling and prevent autoimmunity. *Nature* **575**, 366–370 (2019). 31546246 10.1038/s41586-019-1612-6 PMC6856441

[69] Roucourt, B., Meeussen, S., Bao, J., Zimmermann, P. & David, G. Heparanase activates the syndecan-syntenin-ALIX exosome pathway. *Cell Res.* **25**, 412–428 (2015). 25732677 10.1038/cr.2015.29 PMC4387558

[70] Zhou, L.-L. et al. MicroRNA‑143‑3p contributes to the regulation of pain responses in collagen‑induced arthritis. *Mol. Med Rep.* **18**, 3219–3228 (2018). 30066874 10.3892/mmr.2018.9322 PMC6102648

[71] Balaskas, P. et al. MicroRNA Signatures in Cartilage Ageing and Osteoarthritis. *Biomedicines* **11**10.3390/biomedicines11041189 (2023). 10.3390/biomedicines11041189 PMC10136140 37189806

[72] Yang, Z., Wang, J., Pan, Z. & Zhang, Y. miR-143-3p regulates cell proliferation and apoptosis by targeting IGF1R and IGFBP5 and regulating the Ras/p38 MAPK signaling pathway in rheumatoid arthritis. *Exp. Ther. Med.* **15**, 3781–3790 (2018). 29581736 10.3892/etm.2018.5907 PMC5863597

[73] Lopez-Fabuel, I. et al. Aberrant upregulation of the glycolytic enzyme PFKFB3 in CLN7 neuronal ceroid lipofuscinosis. *Nat. Commun.* **13**, 536 (2022). 35087090 10.1038/s41467-022-28191-1 PMC8795187

[74] Zhou, J. et al. Glycerol kinase 5 confers gefitinib resistance through SREBP1/SCD1 signaling pathway. *J. Exp. Clin. Cancer Res.* **38**, 96 (2019). 30791926 10.1186/s13046-019-1057-7 PMC6385389

