# Supplementary material for: Strontium–Alix interaction enhances exosomal miRNA selectively loading in synovial MSCs for temporomandibular joint osteoarthritis treatment
Source: Int J Oral Sci. 2025 Feb 1;17:6. doi: 10.1038/s41368-024-00329-5 (PMC11785994; doi:10.1038/s41368-024-00329-5)
Supplement: Supplementary file 1 — Supplementary Materials [file 41368_2024_329_MOESM1_ESM.pdf]

Supplementary Materials for

Strontium-Alix interaction enhances exosomal miRNA selectively loading in synovial MSCs for temporomandibular joint osteoarthritis treatment

Authors: Wenxiu Yuan [1, 2, 3], Jiaqi Liu [1, 2], Zhenzhen Zhang [1, 2], Chengxinyue Ye [1, 2], Xueman Zhou [1, 2], Yating Yi [1], Yange Wu [1, 2], Yijun Li [1, 2], Qinlanhui Zhang [1, 2], Xin Xiong [1], Hengyi Xiao [2], Jin Liu [2, *], Jun Wang [1, *]

Supplementary Figures

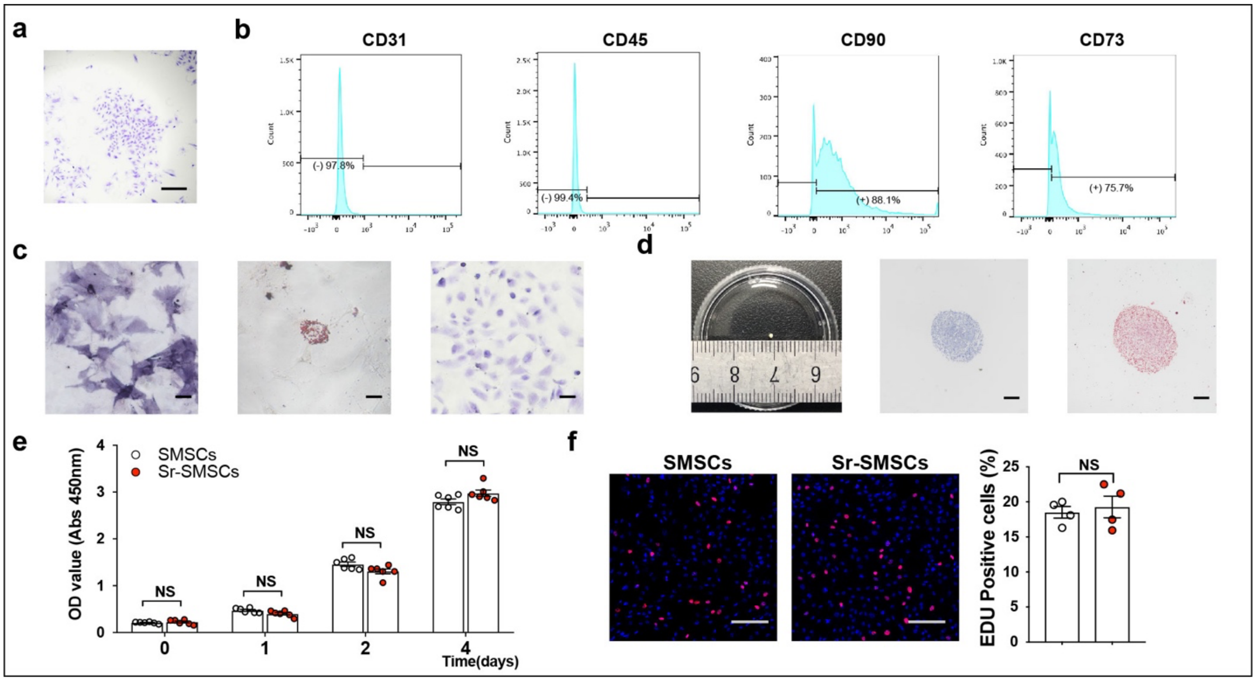

Fig. S1. Characterization of SMSCs and effects of Sr on the activity and proliferation of SMSCs. a Crystal violet staining of cell colony. Scale bars, 100 μm. **b** Flow cytometric analysis of the expression of cell surface markers. **c** Two-dimensional multidirectional differentiation

culture and ALP, Oil Red O, and Toluidine blue staining. Scale bars, 50 μm. **d** Three-dimensional chondrogenic differentiation culture and Safranin-O and Toluidine blue staining. Scale bars, 200 μm. SMSC cell viability determined by **e** CCK-8 assay and **f** EDU assay after indicated treatment. Scale bar, 100 μm.

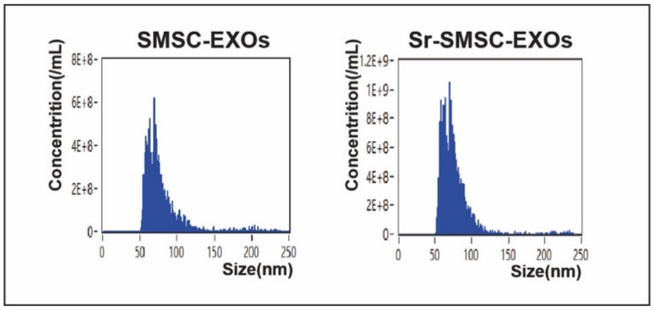

Fig. S2. Size distribution of particles were assessed by nanoparticle tracking analysis.

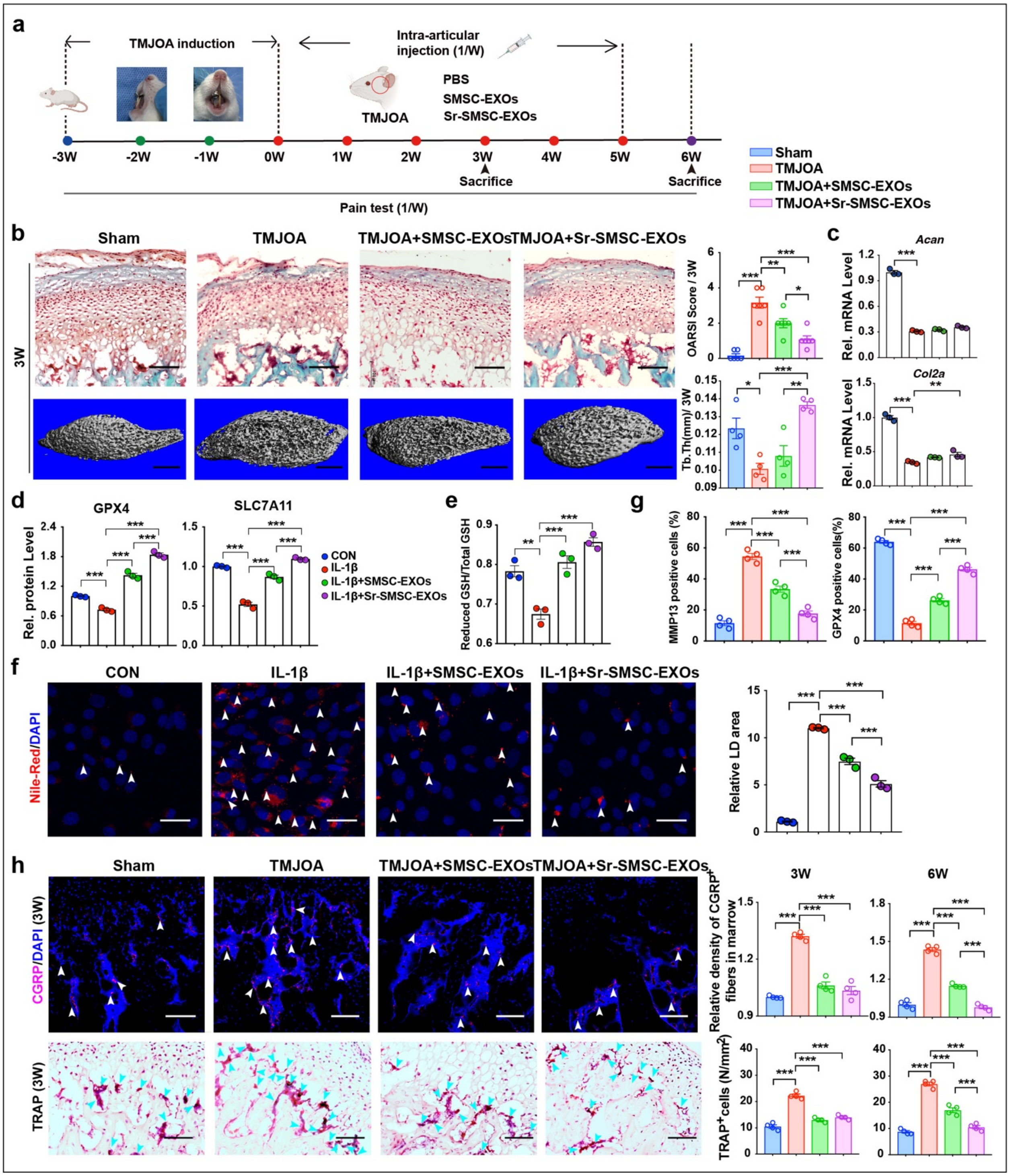

Fig. S3. Superior therapeutic performance of Sr-enhanced SMSC-EXOs in ameliorating TMJOA symptoms in rats. a Schematic model of the time course for establishment of unilateral anterior crossbite (UAC) model of TMJOA in rats treated with exosomes and pain testing. **b**

Safranin-O/Fast green staining, OARSI scoring system in condylar cartilage, 3D reconstruction of the condyles and trabecular thickness (Tb. Th) in subchondral bone at 3 weeks. Scale bar for Safranin-O/Fast green staining and immunofluorescence staining, 100 μm; Scale bar for 3D reconstruction, 1 mm. **c** Quantitative RT-PCR analysis of condylar chondrocytes 48h after indicated treatment. **d** Western blot quantitative analyses of the GPX4 and SLC7A11 in condylar chondrocytes 48h after indicated treatment. **e** Measurement of GSH content in condylar chondrocytes 48h after indicated treatment. **f** Nile red staining of condylar chondrocytes 48h after indicated treatment. Scale bar, 50 μm. Arrowheads indicate lipid droplets. **g** Immunofluorescence staining quantitative analyses of MMP13 and GPX4 of condylar cartilage. **h** Immunofluorescence staining for CGRP and TRAP staining in subchondral bone at 3 weeks and quantitative analyses at 3 and 6 weeks. Scale bar, 100 μm. Arrowhead indicate positive cells. Data are represented as mean ± SEM. * $p < 0.05$, ** $p < 0.01$, *** $p < 0.001$.

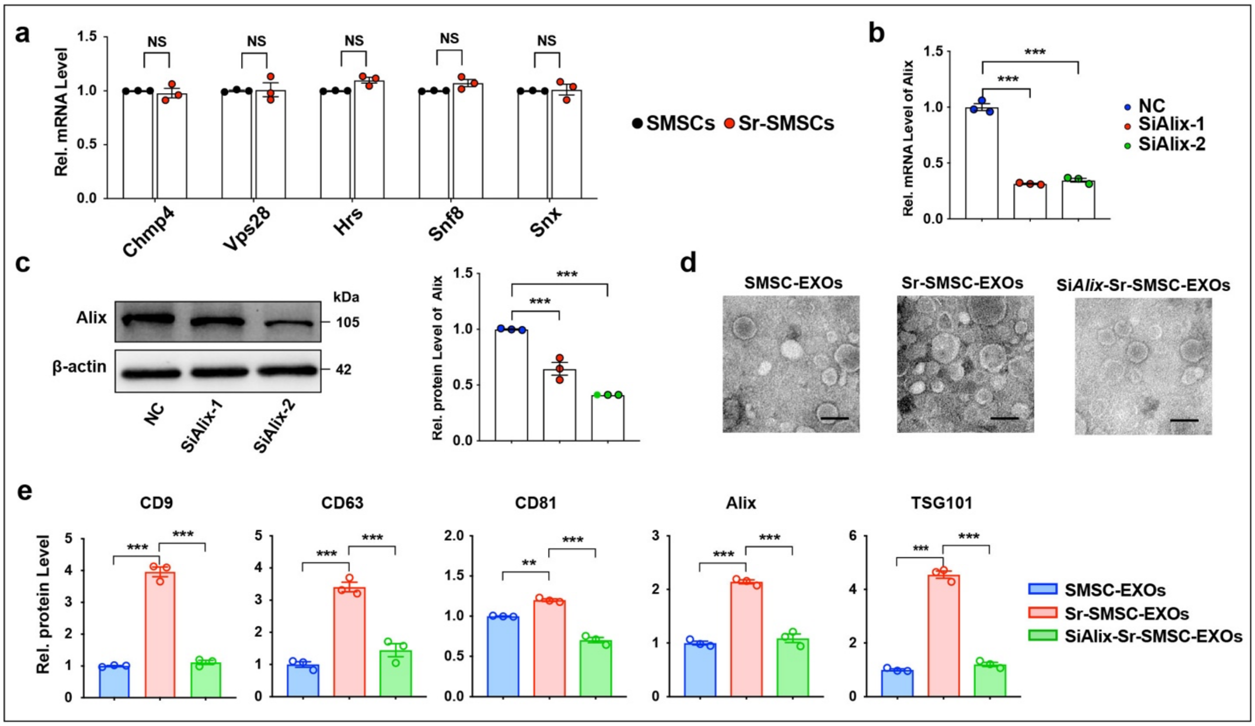

Fig. S4. Increasing Alix by Sr-pretreating SMSCs boosts the yield of EXOs. a Quantitative RT-PCR analyses of exosome formation related genes in SMSCs 48h after indicated treatment. **b** Quantitative RT-PCR analyses of Alix expression in SMSCs after indicated treatment. **c** Western blot quantitative analyses of Alix in SMSCs after indicated treatment. **d** Electron micrographs of exosomes. Scale bars, 200 nm. **e** Western blot quantitative analyses of marker proteins CD9, CD63, CD81, and TSG101 in exosomes. Data are represented as mean ± SEM. * $p < 0.05$, ** $p < 0.01$, *** $p < 0.001$.

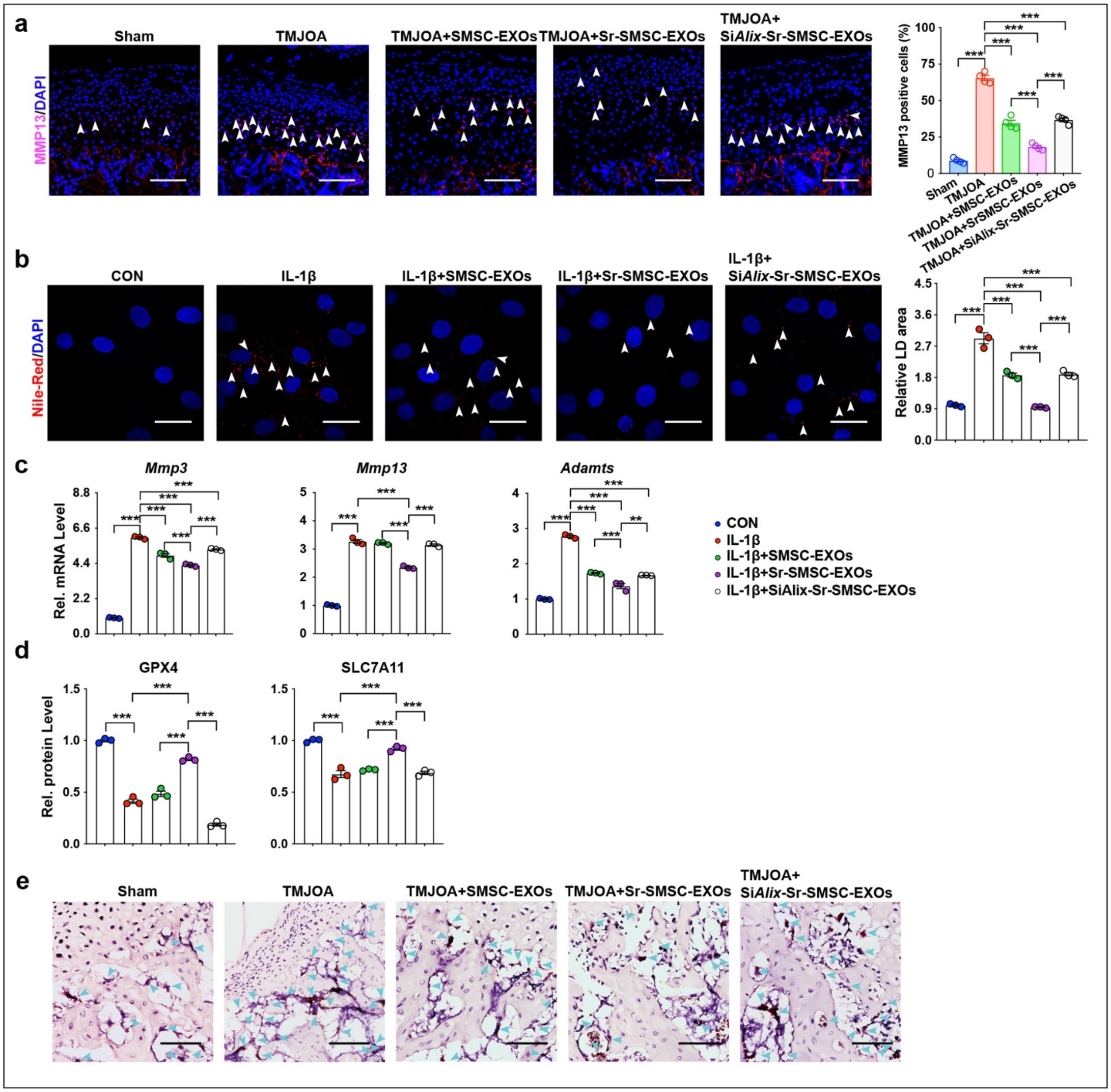

Fig. S5. Alix mediates the effect of Sr on TMJOA therapeutic efficacy of SMSC-EXOs. a Immunofluorescence staining for MMP13 in condylar cartilage at 6 weeks. Arrowheads indicate positive cells. Scale bar, 100 μm. **b** Nile red staining of condylar chondrocytes 48h after indicated treatment. Scale bar, 50 μm. Arrowheads indicate positive cells. **c** Quantitative RT-PCR analyses of *Mmp3, Mmp13,* and *Adamts* in condylar chondrocytes 48h after indicated treatment. **d** Western blot quantitative analyses of the SLC7A11 and GPX4 in condylar chondrocytes 48h after indicated

treatment. **e** TRAP staining in subchondral bone. Scale bar, 100 μm. Arrowheads indicate positive cells. Data are represented as mean ± SEM. * $p < 0.05$, ** $p < 0.01$, *** $p < 0.001$.

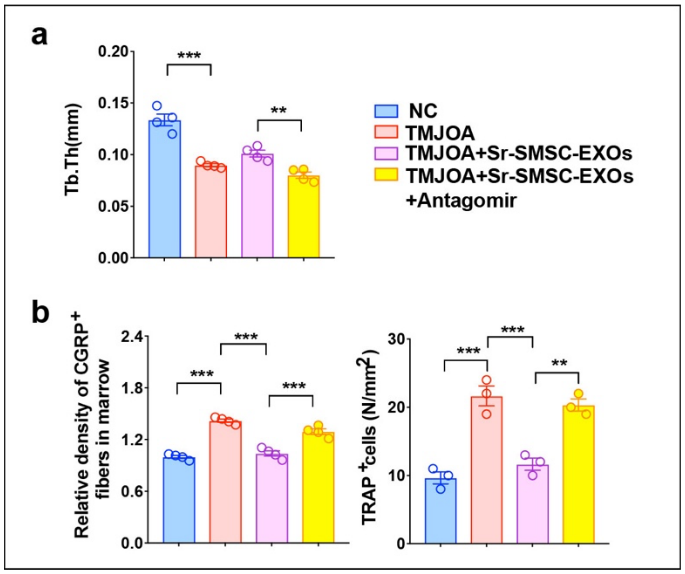

Fig. S6. Upregulation of miR-143-3p in Sr-SMSC-EXOs contributes to their enhanced therapeutic efficacy. a Tb. Th of subchondral bone. **b** Immunofluorescence staining quantitative analyses of CGRP in subchondral bone and TRAP staining quantitative analyses of subchondral bone. Data are represented as mean ± SEM. * $p < 0.05$, ** $p < 0.01$, *** $p < 0.001$.

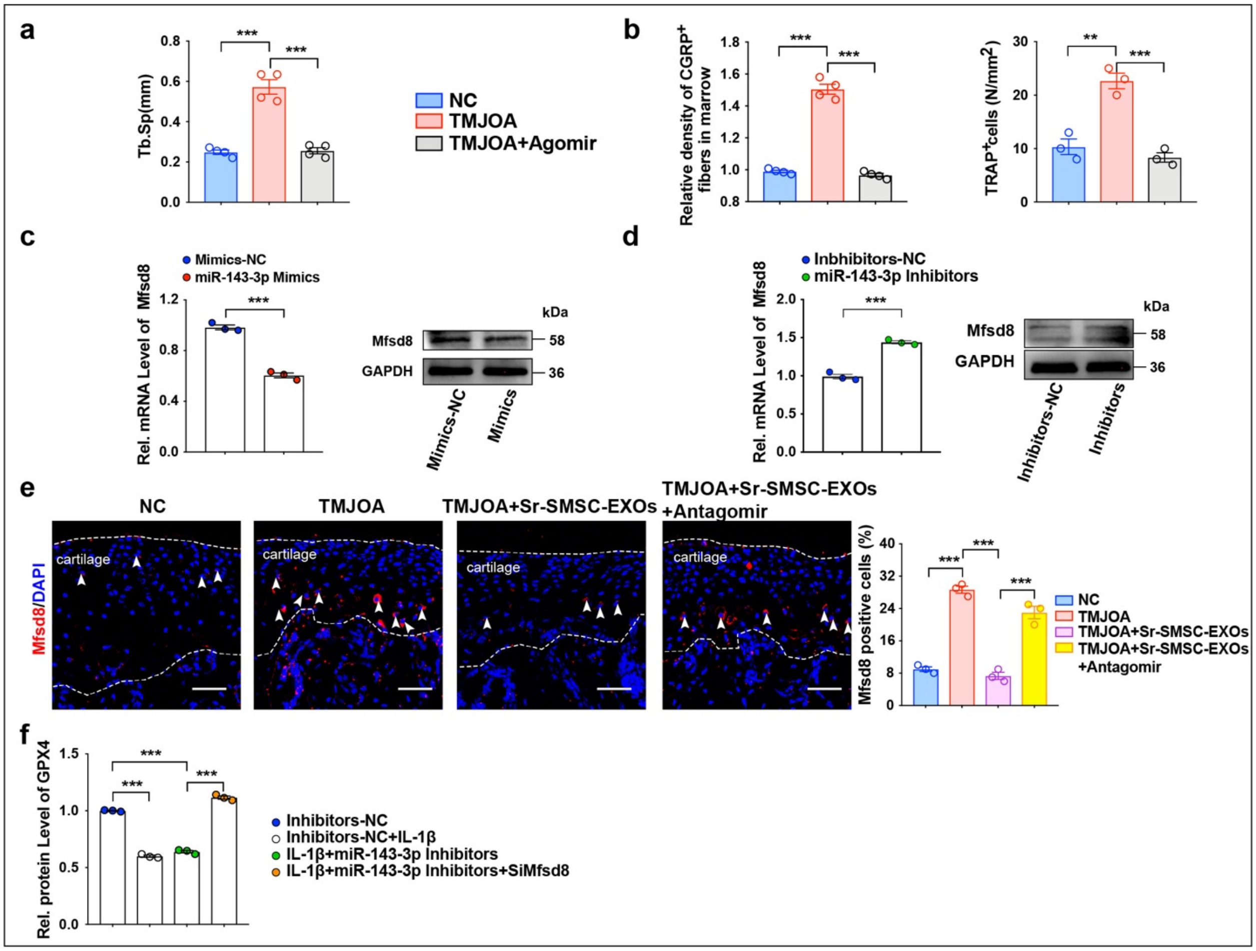

Fig. S7. MiR-143-3p alleviates TMJOA via targeting Mfsd8-mediated chondrocyte ferroptosis. **a** Tb. Sp of subchondral bone. **b** Immunofluorescence staining quantitative analyses of CGRP and TRAP staining in subchondral bone. **c d** Quantitative RT-PCR and western blot analyses of the Mfsd8 in chondrocytes 48h after indicated treatment. **e** Immunofluorescence staining for Mfsd8 of condylar cartilage. Scale bar, 100 μm. Arrowheads indicate positive cells. **f** Western blot quantitative analyses of GPX4 in chondrocytes 48h after indicated treatment. Data are represented as mean ± SEM. *** $p < 0.001$.

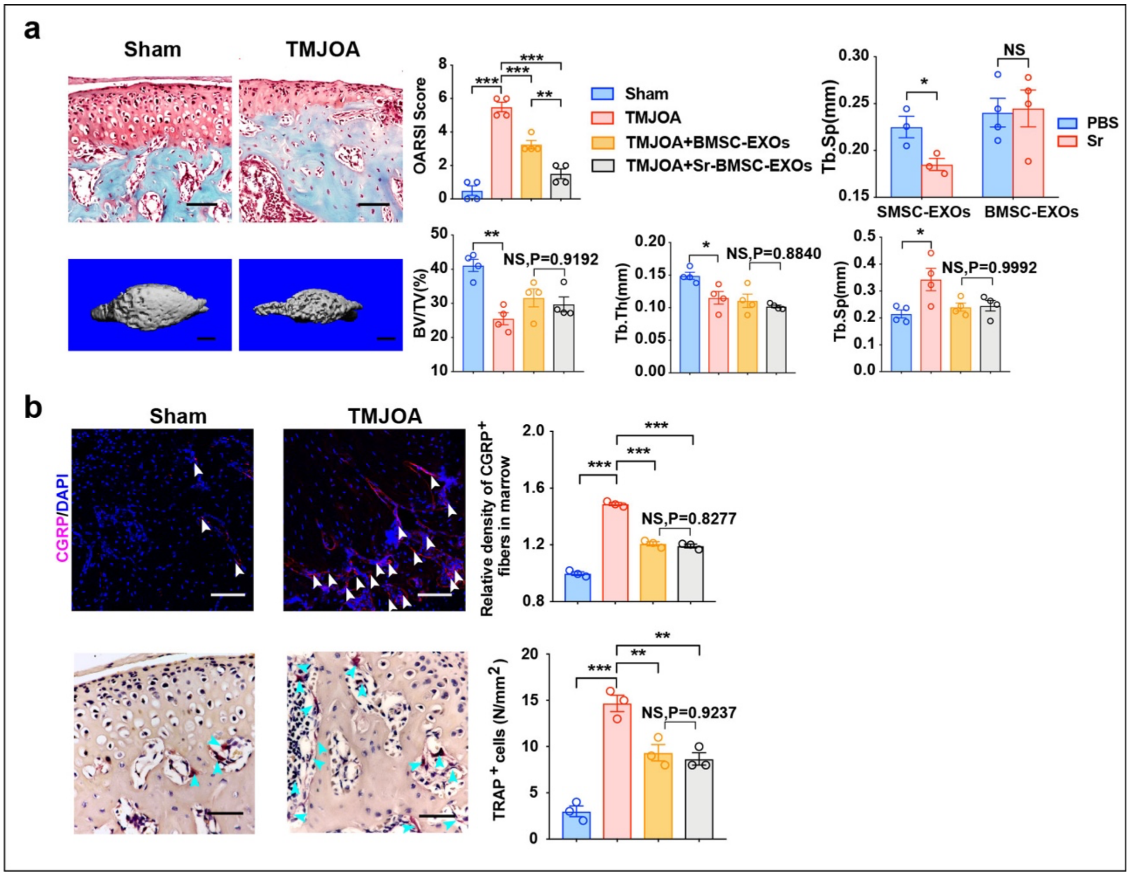

Fig. S8. Synovium niche-derived MSCs, but not bone marrow-derived MSCs, undergo Alix-mediated exosomal miRNA loading upon strontium exposure. a Safranin-O/Fast green staining and OARSI scoring system in condylar cartilage, 3D reconstruction of the condyles and BV/TV, Tb. Sp, and Tb. Th in subchondral bone. Scale bar for Safranin-O/Fast green staining and immunofluorescence staining, 100 μm; Scale bar for 3D reconstruction, 1 mm. **b** Immunofluorescence staining for CGRP and TRAP staining in subchondral bone. Scale bar, 100 μm. Arrowheads indicate positive cells. Data are represented as mean ± SEM, NS, not significant, * $p < 0.05$, ** $p < 0.01$, *** $p < 0.001$.

Supplementary Tables

Table S1. Properties of differentially expressed miRNAs with well-defined functions Sr-SMSC-EXOs versus SMSC-EXOs.

miRNA	Function	Chang	Consistent or not	Rank in SMSC-EXOs	Rank in Sr-SMSC-EXOs
miR-370-3p	harmful	↓	Yes	81	117
miR-363-3p	harmful	↓	Yes	137	293
miR-146a-5p	harmful	↓	Yes	58	110
miR-671	beneficial	↓	No	173	480

Table S2. Properties of differentially expressed miRNAs with well-defined functions (Si*Alix*-Sr-SMSC-EXOs versus (Sr-SMSC-EXOs versus SMSC-EXOs)).

miRNA	Function	Chang	Consistent or not	Rank in Sr-SMSC-EXOs	Rank in Si*Alix*-Sr-SMSC-EXOs
miR-146a-5p	harmful	↑	Yes	110	68
miR-26a-5p	harmful	↑	Yes	8	6
miR-140-3p	beneficial	↓	Yes	37	47

Table S3. Sequences of Antagomir and Agomir.

Name	Sequence
Antago-NC	5’-CAGUACUUUUGUGUAGUACAA-3’
Antago-miR-143-3p	5’-UGAGCUACAGUGCUUCAUCUCA-3’
Ago-NC	Sense: 5’-UUCUCCGAACGUGUCACGUTT-3’
	Anti-sense: 5’-ACGUGACACGUUCGGAGAATT-3’
Ago-miR-143-3p	Sense: 5’-UGAGAUGAAGCACUGUAGCUCA-3’
	Anti-sense: 5’-AGCUACAGUGCUUCAUCUCAUU-3’

Table S4. Primers for quantitative RT-PCR.

Gene	Primer Sequence
GAPDH	F 5’- AGACAGCCGCATCTTCTTGT -3’
	R 5’- TGGACACCATACTTGAGCAGAG -3’
MMP3	F 5’- TCTTTCACTCAGCCAATGCT -3’
	R 5’- GGGAGGTCCATAGAGGGATT -3’
MMP9	F 5’- AGCCGGGAACGTATCTGGA -3’
	R 5’- TGGAAACTCACACGCCAGAAG -3’

MMP13	F 5’- GCCCTGAATGGGTATGACAT -3’
	R 5’- GCATGACTCTCACAATGCGA -3’
ADAMTS	F 5’- CATACCCTGCCCACCTAAC -3’
	R 5’- GTTCTCACGCACCTTCCTC -3’
PTGS2	F 5’- ATCCTTGCTGTTCCAACCCA -3’
	R 5’- TCTTGTCAGAAACTCAGGCGTA -3’
GPX4	F 5’- CCATTCCCGAGCCTTTCAACC -3’
	R 5’- TCGGTTTTGCCTCATTGCGA -3’
SLC7A11	F 5’- TGCTGCCTACACAAAGACGTT -3’
	R 5’- CGCCTTGCCCTTTAAGTATTCACC -3’
Alix	F 5’- ATCGGATCTATGGTGGGCTAA -3’
	R 5’- CCTCACTGTTAGACTGCTTCATT -3’
Mfsd8	F 5’-CCGATCATACATTGCCGGTG -3’
	F 5’- F 5’-GGCATGTGCTCGTATTAGCC -3’
miR-146a-5p	5’- GCAGAGTGAGAACTGAATTCCA -3’
miR-191a-5p	5’- GAGCAACGGAATCCCAAAAG -3’
miR-1298	5’- CGAGTTCATTCGGCTGTCCA -3’
miR-219a-1-3p	5’- CGAGAGAGTTGCGTCTGGAC -3’
miR-143-3p	5’- GCCGATGAGATGAAGCACTGT -3’
miR-26a-5p	5’- GGCAGGTTCAAGTAATCCAGGA -3’
miR-92a-3p	5’- AGCAGGTATTGCACTTGTCCC -3’
miR-140-3p	5’- CGGCAGTACCACAGGGTAGAA -3’
U6	5’- CCATTCCCGAGCCTTTCAACC -3’

Table S5. RNAi sequence.

Gene	Sequence
Alix	F 5’- GACUUGGUUAACAGAUCAATT -3’
	R 5’- UUGAUCUGUUAACCAAGUCTT -3’
Mfsd8	F 5’- CCAAUUGCUUGUACGCAUA -3’
	F 5’- UAUGCGUACAAGCAAUUGG -3’

Table S6. Sequences of Mimics and Inhibitors.

Name	Sequence
Mimics-NC	Sense: 5’-UUCUCCGAACGUGUCACGUTT-3’
	Anti-sense: 5’-ACGUGACACGUUCGGAGAATT-3’
miR-143-3p mimics	5’-UGAGAUGAAGCACUGUAGCUCA-3’
Inhibitors-NC	5’-CAGUACUUUUGUGUAGUACAA-3’
miR-143-3p inhibitors	5’-UGAGCUACAGUGCUUCAUCUCA-3’
